# Optimal decision-making in relieving global high temperature-related disease burden by data-driven simulation

**DOI:** 10.1016/j.idm.2024.03.001

**Published:** 2024-03-19

**Authors:** Xin-Chen Li, Hao-Ran Qian, Yan-Yan Zhang, Qi-Yu Zhang, Jing-Shu Liu, Hong-Yu Lai, Wei-Guo Zheng, Jian Sun, Bo Fu, Xiao-Nong Zhou, Xiao-Xi Zhang

**Affiliations:** a School of Global Health, Chinese Center for Tropical Diseases Research, Shanghai Jiao Tong University School of Medicine, Shanghai, People's Republic of China; b Institute of One Health, Shanghai Jiao Tong University, Shanghai, People's Republic of China; c School of Public Health, Shanghai Jiao Tong University School of Medicine, Shanghai, People's Republic of China; d School of Data Science, Fudan University, Shanghai, People's Republic of China

**Keywords:** High temperature-related diseases, Data-driven simulation, Optimal intervention, Disease burden, Graph neural network, Global warming

## Abstract

The rapid acceleration of global warming has led to an increased burden of high temperature-related diseases (HTDs), highlighting the need for advanced evidence-based management strategies. We have developed a conceptual framework aimed at alleviating the global burden of HTDs, grounded in the One Health concept. This framework refines the impact pathway and establishes systematic data-driven models to inform the adoption of evidence-based decision-making, tailored to distinct contexts. We collected extensive national-level data from authoritative public databases for the years 2010–2019. The burdens of five categories of disease causes – cardiovascular diseases, infectious respiratory diseases, injuries, metabolic diseases, and non-infectious respiratory diseases – were designated as intermediate outcome variables. The cumulative burden of these five categories, referred to as the total HTD burden, was the final outcome variable. We evaluated the predictive performance of eight models and subsequently introduced twelve intervention measures, allowing us to explore optimal decision-making strategies and assess their corresponding contributions. Our model selection results demonstrated the superior performance of the Graph Neural Network (GNN) model across various metrics. Utilizing simulations driven by the GNN model, we identified a set of optimal intervention strategies for reducing disease burden, specifically tailored to the seven major regions: East Asia and Pacific, Europe and Central Asia, Latin America and the Caribbean, Middle East and North Africa, North America, South Asia, and Sub-Saharan Africa. Sectoral mitigation and adaptation measures, acting upon our categories of Infrastructure & Community, Ecosystem Resilience, and Health System Capacity, exhibited particularly strong performance for various regions and diseases. Seven out of twelve interventions were included in the optimal intervention package for each region, including raising low-carbon energy use, increasing energy intensity, improving livestock feed, expanding basic health care delivery coverage, enhancing health financing, addressing air pollution, and improving road infrastructure. The outcome of this study is a global decision-making tool, offering a systematic methodology for policymakers to develop targeted intervention strategies to address the increasingly severe challenge of HTDs in the context of global warming.

## List of abbreviations

HTDs: high temperature-related diseases
GNN: Graph Neural Network
GHG: greenhouse gas
GBD: Global Burden of Disease
HVI: Heat Vulnerability Index
UFCS: urban framework for climate service
WMO: World Meteorological Organization
WHO: World Health Organization
HHWS: Heat Health Warning Systems
IPCC: Intergovernmental Panel on Climate Change
AFOLU: agriculture, forestry, and other land use
CMNND: communicable, maternal, neonatal, and nutritional diseases
FAO: Food and Agriculture Organization
GHDx: Global Health Data Exchange
RMSE: Root Mean Squared Error
MAE: Mean Absolute Error
MAPE: Mean Absolute Percentage Error
RMSPE: Root Mean Squared Percentage Error
CHaRT: Climate and Health Risk Tool

## 1 Introduction

Human activities, chiefly through greenhouse gas (GHG) emissions, have steadily contributed to global warming and climate change. The decade spanning 2011 to 2020 saw a global surface temperature increase of 1.1 °C over the 1850–1900 baseline, associated with an uptick in extreme weather events such as heatwaves and droughts (IPCC, 2023). Research has confirmed the detrimental effects of intense environmental heat and associated stress, leading to increased rates of mortality and morbidity (An der Heiden et al., 2020; Morefield et al., 2018; Schulte et al., 2021; Winklmayr et al., 2022; Zhou et al., 2022), negative pregnancy outcomes (Barreca & Schaller, 2020; Bekkar et al., 2020), impaired mental health (Clayton, 2021; Marazziti et al., 2021; Palinkas & Wong, 2020), and impacts on workforce productivity (Dasgupta et al., 2021; Lee et al., 2018). According to the Global Burden of Disease (GBD) study findings (Burkart et al., 2021), high temperatures were responsible for 0.54% of death and 0.46% of Disability-Adjusted Life Years (DALY) in 2019, with notable regional and socio-economic inequities (Song et al., 2021). Historical interventions primarily focused on human health, such as air conditioning (Kouis et al., 2021) and heat exposure reduction (Mallen et al., 2022), have been implemented, but temperature variations induce other significant ecological changes, affecting complex factors at the intersection of human-animal-environment interactions. These ecological changes affect the speed of pathogen transmission (Samuel et al., 2016), the activity of disease vectors (Shapiro et al., 2017), and the dispersion of pollutants (Kinney, 2018). Additionally, these factors interplay with various socioeconomic and environmental effects, including meteorological variables (Fang et al., 2023), urban heat island effects (Heaviside et al., 2017; Ho et al., 2023), demographic aging (Huang et al., 2022; Xing et al., 2022), and air quality (Hu et al., 2022; Xing et al., 2022). Crucially, such interactions influence the emergence and progression of diseases and other health-related incidents. To address these complex interactions in high temperature-related diseases (HTDs), the One Health approach emphasizes the intricate influences between human, animal, and environmental domains, calling for greater consideration of broader socioeconomic factors (Jesudason, 2022, Zhang et al., 2022, Zhang et al., 2023).

In response to the escalating challenge of HTDs, the advancement of evidence-based management strategies has become an eminent frontier in public health and environmental science. Recent research underscores a variety of potential strategies aimed at mitigating the detrimental impacts of elevated temperatures on human health. Such strategies span from geospatial analytics and predictive modeling to the deployment of real-time monitoring systems and community-led initiatives. Anchored in environmental science and epidemiology, these studies exploit comprehensive data on weather trends, at-risk population, and healthcare capabilities to inform proactive and targeted responses. Presently, research efforts to combat HTDs fall into three broad categories: risk assessment, intervention evaluation, and the optimization of decision-making processes. Risk assessment utilizes both historical and predictive data to craft vulnerability indices (Nayak et al., 2018; Turek-Hankins et al., 2020) and employs visualization technologies (Hess et al., 2023) to gauge the likelihood and severity of heatwaves with a particular focus on vulnerable populations and areas. Evaluation of interventions measures the efficacy and cost-effectiveness of heat mitigation tactics and public health initiatives, such as green infrastructure (Chen et al., 2014; Choi et al., 2022; Sailor et al., 2021), Heat Health Prevention Plans (HHPP) (Martínez-Solanas & Basagaña, 2019), and cool roofs (Macintyre & Heaviside, 2019). Some studies also employ quasi-experimental designs to compare intervention outcomes, enhancing strategy effectiveness assessments (Li et al., 2016). Optimal decision-making focuses on strategic resource distribution and real-time response management through decision science and systems analysis, facilitating informed decisions under uncertainty. Despite being a relatively new area within HTD research, innovative approaches are emerging; for example, system dynamics models analyzing short versus long-term interventions on mortality within an Urban Climate Service Framework (Liu et al., 2020), an evaluation of mitigation options and combinations to reduce ambient temperatures (Haddad et al., 2020), an epidemiological survey to identify possible adaptation measures among older adults (Fujimoto et al., 2023), and an interactive web application to support smart tree-planting in Boston (Werbin et al., 2020).

Furthermore, current decision analysis modeling and optimization technologies in the field of public health can be broadly divided into two categories. The first includes traditional decision science methods such as constrained optimization (Earnshaw, 2018), Markov models, cost-benefit analyses (Gupta et al., 2020), and system dynamics modelling (Currie et al., 2018), among others. These methods rely on structured frameworks for evaluating and making decisions based on a variety of criteria and constraints. However, they may lack the flexibility to automatically adapt to new data or complex relationships within data. The second category comprises computational optimization algorithms, with a focus on data-driven technologies within the broader scope of artificial intelligence (AI) and machine learning (ML). This category extends beyond traditional statistical methods such as generalized linear regression to include more advanced machine learning algorithms like decision trees, random forests, support vector machines, and neural network, including deep learning techniques (Adiga et al., 2020). These methods offer the advantage of handling large volumes of data and automatically identifying complex patterns that may not be evident through traditional methods. Precedents in contemporary research include machine learning-based COVID-19 clinical decision support (Karthikeyan et al., 2021) and online decision support systems for prostate cancer using long short-term memory (LSTM) artificial neural network (ANN) models (Koo et al., 2020), which both highlight the potential advantages of data-driven technologies in decision-making for health interventions. Especially when dealing with interdisciplinary topics such as the impact of climate change on health, data-driven approaches can enhance the assessment and prediction of future high-temperature scenarios with minimal assumptions, thereby promoting more effective risk management and resource allocation.

Despite significant advancements in understanding and addressing the health impacts of high temperatures, notable gaps persist. Firstly, there is a noticeable lack of literature employing systematic thinking and the One Health concept in developing policy simulation tools for HTDs. This shortfall spans from analyzing impact factors to strategizing intervention measures. Discussion on intervention strategies predominantly centers around climate change adaptation, often limited to standalone actions without a holistic examination of integrated, multi-faceted strategies. Secondly, despite the proven effectiveness of data-driven modeling in uncovering the complex dynamics of health hazards and risks, its application in HTD mitigation remains underutilized (Ebi et al., 2021, Song et al., 2021). These advanced modeling techniques, now commonplace in climate science for identifying critical processes in global temperature and sea level changes (Song et al., 2023), predicting global warming milestones (Diffenbaugh & Barnes, 2023), and issuing immediate regional heatwave alerts (Li et al., 2023), have yet to be fully integrated into HTD decision-making processes. Finally, the scope of current research is limited. Current studies primarily focus on urban scales without national or global implications. Although nations play pivotal roles within the public health climate policy framework, melding diverse national strategies into a unified, optimal decision-making model is difficult. Consequently, crafting adaptable, scalable intervention frameworks that cater to the varied conditions across countries is a significant opportunity.

In this study, we developed a framework for optimal decision-making to alleviate the global burden of HTDs, grounded in the One Health approach. We then designed and evaluated eight data-driven models for simulation purposes. Utilizing a real-world database, we proposed optimized decision-making packages, tailored for various scenarios across different geographical regions.

## 2 Methods

### 2.1 Pathway construction

#### 2.1.1 Overall design

**Fig. 1:**
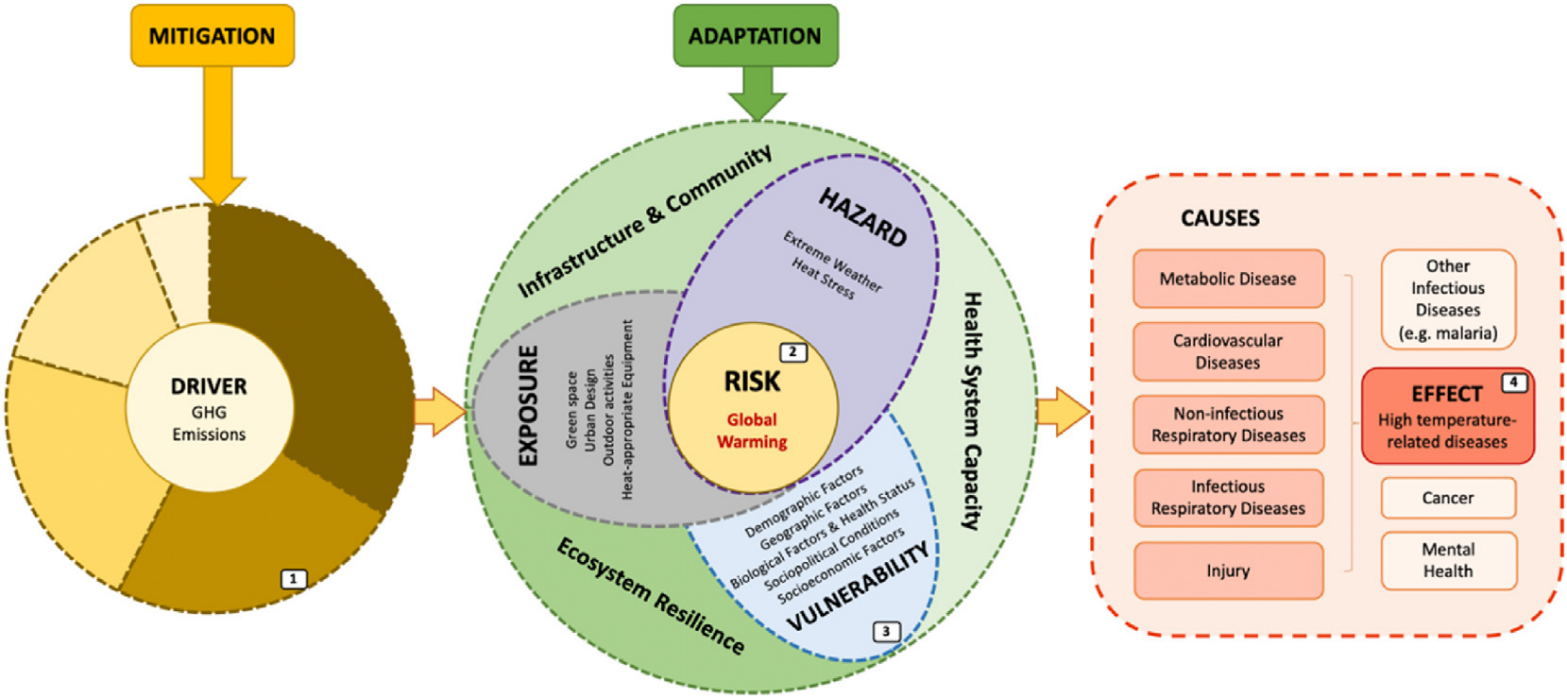
Conceptual Framework for Optimal Decision-making in Relieving Global HTD Burden based on One Health Approach.

Drawing on comprehensive literature research (see Appendix A), we synthesized the impact frameworks and pathway diagrams related to climate change's effects on health, as established by the WHO and the Intergovernmental Panel on Climate Change (IPCC), among others (Ara Begum et al., 2022; Berry et al., 2018; Chiabai et al., 2018; Kjellstrom & McMichael, 2013; Phalkey et al., 2016; World Health Organization, 2021). Within these frameworks, we developed a conceptual framework aimed at facilitating optimal decision-making to mitigate the global burden of HTDs grounded in the One Health approach (Fig. 1.). This framework serves as the basis for pathway construction, delineation of system boundaries, and identification of critical elements. It consists of three main systems: the GHG emission drive system, the global warming risk system, and the specific high temperature-attributable burden system. This integrated approach combines factors related to global warming's impact on HTDs and includes interdisciplinary and cross-sectoral strategies for climate change adaptation and mitigation. Central of this framework is the global warming risk, influenced by risk determinants: hazard, vulnerability, and exposure, while considering feedback mechanisms, cascades, non-linear dynamics, and the potential for unforeseen events (Ara Begum et al., 2022).

[1] The sectoral chart segments illustrate direct GHG emissions by sector for 2019, including construction, transport, agriculture, forestry, and other land use, industry, and energy systems (Dhakal et al., 2022).
[2] Global warming risks are depicted in terms of hazard, exposure, and vulnerability, as outlined in Chapter 1 of the IPCC's Climate Change 2022 report (Ara Begum et al., 2022).
[3] Here, ‘vulnerability’ encompasses demographic, geographic, biological, and health factors, along with sociopolitical and socioeconomic conditions, as defined by the WHO (World Health Organization, 2021).
[4] Utilizing data from the GBD Study 2019, the initial analysis attributed to high temperatures covered metabolic, cardiovascular, non-infectious respiratory, infectious respiratory diseases and injuries, but may have overlooked cancer, mental health, and other infectious diseases (Burkart et al., 2021; Song et al., 2021).

#### 2.1.2 Subsystems design

In the established framework, the primary drivers of global warming stem from GHG emissions across various sectors. In 2019, the total global GHG emissions amounted to 59 GtCO_2_-equivalent, with contributions from the energy sector (34%), industry (24%), agriculture, forestry, and other land use (AFOLU) (22%), transport (15%), and buildings (6%) (Dhakal et al., 2022). Mitigation strategies are thus tailored to these sectors.

The concept of hazard in this context includes heat stress, characterized by both long-term average temperature increases and short-term heatwaves, as well as extreme weather events. Exposure considers elements such as green spaces, urban design, outdoor activities, and heat-appropriate equipment. Vulnerability is determined by demographic, geographic, biological and health status, sociopolitical conditions, and socioeconomic factors (World Health Organization, 2021). Adaptation strategies fall into three primary categories: infrastructure and community initiatives, ecosystem resilience, and health system strengthening.

To support the modeling and analysis of these factors and corresponding intervention strategies, we revised the categorization of causes in the GBD related to HTDs. The original level 1 causes encompass communicable, maternal, neonatal, and nutritional diseases (CMNND), non-communicable diseases, and injuries. For this study, we divided these into metabolic diseases, cardiovascular diseases, non-infectious respiratory diseases, infectious respiratory diseases, and injuries. Details of this reclassification are available in Appendix B. Cancer, mental illness, other infectious diseases, and other potential HTD causes not yet included in the GBD estimates were excluded from our analysis.

### 2.2 Models specification

#### 2.2.1 Indicator selection

Building on the previously outlined framework, we conducted thorough searches and gathered public datasets extensively. Following a previously developed method (Zhang et al., 2022), indicators were chosen based on their relevance, authoritative sources, accessibility, completeness, timeliness, comparability, and availability of country-level data. The model integrates a total of 37 variables reflecting each subsystem's indicators within the optimal decision-making framework for HTD burden alleviation. Out of these, 31 variables are input variables, including 11 influence factors, 8 key variables, and 12 intervention variables. There are also 5 intermediate outcome variables representing the disease burden of cardiovascular diseases, infectious respiratory diseases, injuries, metabolic diseases, and non-infectious respiratory diseases, plus an outcome variable for the overall HTD burden.

#### 2.2.2 Data sources and processing

We compiled data from 2010 to 2019 for various indicators, primarily sourced from authoritative databases including the WHO, Food and Agriculture Organization of the United Nations (FAO), Global Health Data Exchange (GHDx), and Our World in Data. Some data were calculated based on these authoritative sources.

To ensure a comprehensive and robust analysis, we developed strategies for effective missing data management. Given the reliance on the GBD of HTDs as the primary outcome variable, countries lacking this data were excluded. For the other 31 variables, a 15% threshold for missing data was set to determine exclusion or imputation; countries or regions beyond this threshold were omitted to preserve data integrity. For missing data within this limit, we applied linear interpolation for contiguous missing values and used the country or region's average for isolated missing data points, resulting in 93 countries being included in the model. Appendix C details all variables, including their units, sources, and descriptive statistics.

Significant differences in scale between variables prompted data rescaling, including all 31 input variables and the 5 intermediate outcome variables using min-max normalization between 0 and 1.

#### 2.2.3 Model screening

To initiate our model screening, we compiled a diverse set of eight models, each developed through distinct methodologies. The models span a variety of techniques:

Linear regression assumes a linear relationship between the predictors and the outcome. Decision tree regression (Gordon et al., 1984) uses a tree-like structure for prediction. Support vector regression identifies the optimal hyperplane in a high-dimensional space to facilitate accurate predictions. Adaboost (Drucker, 1997) and Random Forest (RF) (Breiman, 2001) are ensemble learning algorithms aimed at boosting regression model accuracy. XGBoost (Chen & Guestrin, 2016) and XGBoost Random Forest utilize Gradient Boosting Trees to iteratively refine decision tree performance. Graph Neural Network captures system mechanisms effectively by utilizing graph structures. For detailed descriptions of each model, see Appendix D.

Our experiments were conducted on a Linux server equipped with an Intel(R) Xeon(R) CPU Silver 4210 @2.20 GHz and 251G RAM, running Python 3.7.6 with a RTX 2080 GPU. We divided the data into training and test sets with a 70%/30% split.

#### 2.2.4 Model performance evaluation

Model performance was assessed across three dimensions: actual error, relative error, and correlation. Actual error was measured using Root Mean Squared Error (RMSE) and Mean Absolute Error (MAE), while relative error employed Mean Absolute Percentage Error (MAPE) and Root Mean Squared Percentage Error (RMSPE). Correlation analysis was based on the coefficient of determination (R^2^).

A model with low actual and relative errors, coupled with an R^2^ value close to 1, indicates high accuracy in capturing the relationship between input factors and GBD values. Our goal was to identify a model excelling across these performance metrics.

For an in-depth explanation of these metrics and additional evaluation criteria, please refer to Appendix D.

### 2.3 Data-driven simulation

The intervention variables are adjusted based on predetermined rules, and correspond to the output of optimal interventions and their respective contributions.

#### 2.3.1 Intervention variables

We developed a suite of twelve distinct intervention measures to address mitigation strategies in various sectors and adaptation measures within our subcategories: Infrastructure & Community, Ecosystem Resilience, and Health System Capacity. These interventions were organized as detailed in Table 1.

**Table 1:** Categories and subcategories of intervention measures.

Interventions	Category	Subcategory
fertilizer use	mitigation	agricultural sector
livestock feed	mitigation	agricultural sector
manure management	mitigation	agricultural sector
energy intensity	mitigation	energy sector
low-carbon energy production	mitigation	energy sector
plant cover	mitigation	land use change and forestry
air pollution	adaptation	ecosystem resilience
road infrastructure construction	adaptation	infrastructure & community
health care delivery	adaptation	health system capacity
health financing	adaptation	health system capacity
pneumococcal vaccines	adaptation	health system capacity
sanitation	adaptation	health system capacity

For each intervention measure, we fine-turned the parameters according to their specified directions, timing, and intensity to assess the impact on the five cause categories and the overall HTD burden for the year 2019. Interventions were adjusted uniformly in the direction anticipated to reduce the disease burden. The timeframe for these interventions spanned from 2015 to 2019, with an annual intensity set at 4%, reflecting the pattern of real-world policy implementations. Our scope does not include an economic analysis of the interventions, which warrants further investigation in subsequent research.

#### 2.3.2 Optimal decision-making and contribution calculation

The twelve intervention measures can be combined in a total of $\sum_{i=1}^{12}\left(\begin{array}{l} 12\\ i \end{array}\right)$ potential intervention packages. Each package is evaluated against the possible post-intervention HTD burden for 2019. An "optimal" intervention package is defined as the one resulting in the most significant disease burden reduction, with the analysis segmented by the seven regions.

Initially, we identified the optimal package for each disease category and calculated the individual contributions of interventions within these packages. For example, if the optimal package contains three interventions – A, B, and C – resulting in a 2019 disease burden of ‘abc’, then the individual contributions are determined as follows: assuming that interventions BC, AC, and AB result in disease burdens ‘bc’, ‘ac’, and ‘ab’, respectively, the unnormalized contribution for A is a=(bc-abc)/abc, for B it is b=(ac-abc)/abc, and for C it is c=(ab-abc)/abc. The normalized contributions are then calculated as a/(a+b+c), b/(a+b+c), and c/(a+b+c), respectively. It should be noted that based on existing knowledge, the effects of four interventions-air pollution, pneumococcal vaccines, sanitation, and road infrastructure construction are disease-specific, leading to fewer than 4095 valid intervention package combinations.

Subsequently, we pursued the optimal interventions for the combined overall HTD burden, using the same calculation methodology.

#### 2.3.3 Sensitivity analysis

Sensitivity analyses were conducted to assess the influence of varying intervention intensities on the model outcomes. The intervention intensities were adjusted from the base of 4% per annum (cumulating to 20% over five years) to alternative scenarios of 2% per annum (10% over five years) and 6% per annum (30% over five years) to gauge the extent of disease burden reduction under uncertainty in intensity.

## 3 Results

### 3.1 The pathway of decision-making modeling in relieving global HTD burden

**Fig. 2:**
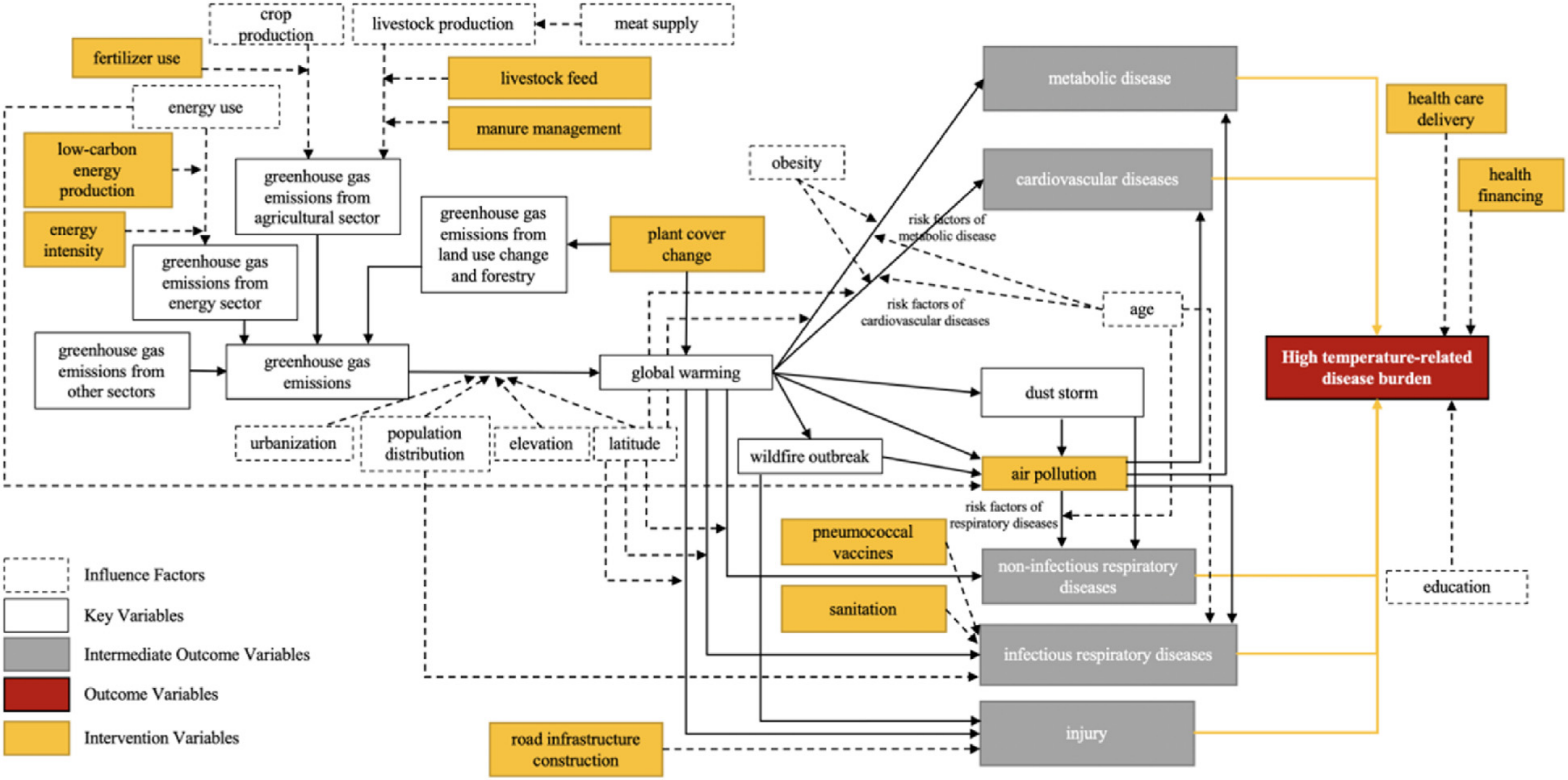
Variable Pathway of Decision-making Modeling in Relieving Global HTD Burden based on One Health Approach. Each arrow indicates a potential influence within the system. The interventions, depicted in yellow, were strategically designed to mitigate various sectors, while the intermediate outcomes, shown in gray, represent the five distinct disease causes. The cumulative impact on HTD burden, the primary outcome of interest, is highlighted in red. Notably, interventions in health care delivery, health financing, and education influence the overall HTD burden through the five disease categories.

Using our framework and findings, we further elucidated the pathway through which climate change affects HTDs, shaping the indicator structure for our model (Fig. 2.). Specifically, greenhouse gas emissions from four sectors contribute to temperature changes, along with variables such as urbanization levels (Chung et al., 2004; Ouyang et al., 2022), population distribution, and geographical factors like altitude and latitude. These temperature rises directly impact the burden of five categories of diseases and influence the climate system, leading to dust storms (De Sario et al., 2013; Johnston et al., 2011), wildfire outbreaks (Ebi, Capon, et al., 2021; Johnston et al., 2011; Shaposhnikov et al., 2014), and related increases in air pollution (De Sario et al., 2013; Patz et al., 2014; J. Song et al., 2021). The literature also underscores air pollution's potential effects on metabolic (Burkart et al., 2022; Chen et al., 2021), cardiovascular (Franklin et al., 2015), and respiratory diseases (Guan et al., 2016; Troeger et al., 2018). Consequently, the HTD burden is a cumulative measure of these disease categories.

Other factors also play a role in HTDs. Latitude (Burkart et al., 2021; Chen et al., 2018; Song et al., 2021) and population density (Song et al., 2021; Zhang et al., 2019) may affect infectious respiratory diseases. Obesity is linked to metabolic and cardiovascular diseases (Clearfield et al., 2014; Hajat et al., 2017; Speakman, 2018), while age influences metabolic, cardiovascular and cerebrovascular diseases, as well as infectious and non-infectious respiratory diseases (Benmarhnia et al., 2016; Chen et al., 2018; Meade et al., 2020; Song et al., 2021; Zhang et al., 2019). Pneumococcal vaccines (Drijkoningen & Rohde, 2014; Zhou et al., 2016) and sanitation (Andrade et al., 2021; Scheelbeek et al., 2021) impact infectious respiratory diseases. Health care coverage (Setoguchi et al., 2022; Sherman et al., 2023; Song et al., 2021), health financing (World Health Organization, 2015), and education (Benmarhnia et al., 2016; Chen et al., 2018) jointly influence the five outcome categories.

### 3.2 Selecting decision-making models for relieving global HTD burden

An examination of the eight models' test outcomes is presented in Table 2. The GNN exhibited the least actual error, suggesting its predictions closely mirror reality. GNN also outperformed other models in terms of relative error, indicating its predictions are comparatively close to actual values. With the highest R^2^ value, GNN's predictions are shown to have a strong correlation with the true values. See Appendix E for more details.

**Table 2:** The comparison of the eight models.

Model	RMSE	MAE	MAPE	RMSPE	R^2^
Linear Regression	0.8349	0.6118	0.6562	0.8482	−0.8601
Decision Tree Regression	0.6412	0.4219	0.3449	0.4851	−0.0969
Support Vector Regression	0.5699	0.3055	0.2159	0.2890	0.1333
Adaboost	0.4918	0.3183	0.2504	0.3238	0.3547
Random Forest	0.4978	0.3206	0.2648	0.3483	0.3388
XGBoost	0.6193	0.3953	0.3231	0.5023	−0.0235
XGBoost Random Forest	0.5587	0.3528	0.2852	0.4212	0.1669
**GNN**	**0.3706**	**0.2447**	**0.1993**	**0.2764**	**0.6334**

In summary, the results suggest that GNN is adept at capturing the complex interplay between input variables and the HTD outcome variable, making it the preferred model for simulation. So, we will proceed with GNN for further intervention experiments and decision optimization.

### 3.3 Optimal decision-making in relieving HTD burden

#### 3.3.1 Optimal interventions for five categories of disease causes

Optimal intervention strategies for cardiovascular diseases include improving low-carbon energy usage, enhancing energy intensity, improving livestock feed, and expanding basic health care delivery coverage. Notably, expanding basic health care delivery coverage is the most significant contributor to reducing the disease burden in North America (57.93%), Europe and Central Asia (38.40%), and Latin America and the Caribbean (32.11%). Additionally, tackling air pollution governs is paramount in mitigating the impact of high temperatures on cardiovascular diseases, especially in South Asia (67.05%), Sub-Saharan Africa (58.34%), Middle East and North Africa (37.34%), and East Asia and Pacific (32.32%).

For infectious respiratory diseases, which carry the highest disease burden among HTDs, the optimal combinations of interventions vary significantly across regions. Vaccination against pneumococcal diseases holds the potential to considerably enhance the situation in the Middle East and North Africa (91.98%) and Sub-Saharan Africa (54.10%). North America (58.45%), along with Latin America and The Caribbean (43.59%), should focus on enhancing their healthcare financing capacities. Meanwhile, addressing air pollution carries higher significance for South Asia (56.16%), as well as East Asia and Pacific (40.47%). Europe and Central Asia, on the other hand, could emphasize improving energy intensity (38.36%) and livestock feed (38.31%).

The optimal interventions for injuries primarily include enhancing low-carbon energy utilization, improving energy intensity, refining livestock feed, expanding basic health care delivery coverage, reducing air pollution, and road infrastructure construction. Among these, expanding basic health care delivery coverage emerges as a pivotal measure for North America (51.24%), Europe and Central Asia (41.88%), East Asia and Pacific (30.88%), and the Middle East and North Africa (28.43%). Latin America and the Caribbean might benefit greatly from boosting low-carbon energy utilization (31.89%) and enhancing road infrastructure (27.39%). Sub-Saharan Africa (36.13%) should also concentrate on enhancing low-carbon energy utilization. Meanwhile, South Asia (38.34%) still faces challenges related to air pollution improvement.

The optimal interventions for metabolic diseases mainly involve enhancing low-carbon energy utilization, expanding basic health care delivery coverage, and reducing air pollution. Among these, increasing low-carbon energy utilization emerges as a higher-contributing measure for North America (81.78%), Europe and Central Asia (44.54%), Latin America and the Caribbean (44.19%), and East Asia and Pacific (34.49%). For South Asia (66.17%), Sub-Saharan Africa (51.17%), and the Middle East and North Africa (39.67%), managing air pollution takes on greater importance.

The optimal interventions for non-infectious respiratory diseases encompass a relatively focused range of measures. For the Middle East and North Africa (90.49%), Latin America and the Caribbean (88.77%), Europe and Central Asia (82.01%), Sub-Saharan Africa (72.30%), and East Asia and Pacific (66.44%), afforestation and reforestation may play a significant role. North America (99.57%) should emphasize energy intensity, while South Asia (60.39%) still needs to address air pollution. Additionally, optimal interventions often include manure management and fertilizer use, which are incorporated across various regions.

**Fig. 3:**
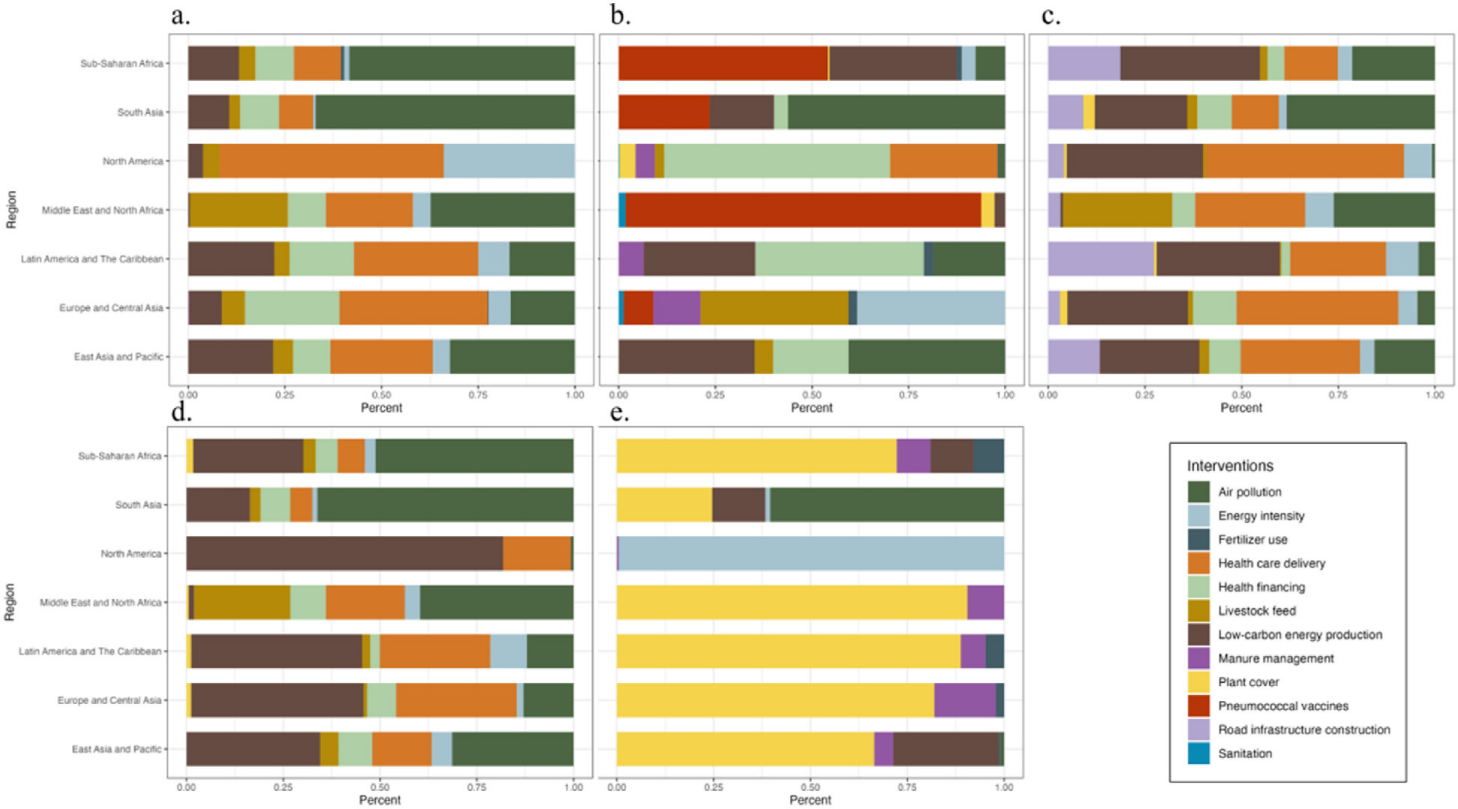
Breakdown of contributions of optimized decision-making packages to relieving HTD burden by regions and causes. (a. Cardiovascular Diseases; b. Infectious Respiratory Diseases; c. Injuries; d. Metabolic Diseases; e. Non-infectious Respiratory Diseases).

The contributions of interventions to the five categories of disease causes for HTD burden reduction under the optimal intervention combination in 2019 are illustrated in Fig. 3. See Appendix F for more details.

#### 3.3.2 Optimal interventions for the aggregated overall HTD burden

**Fig. 4:**
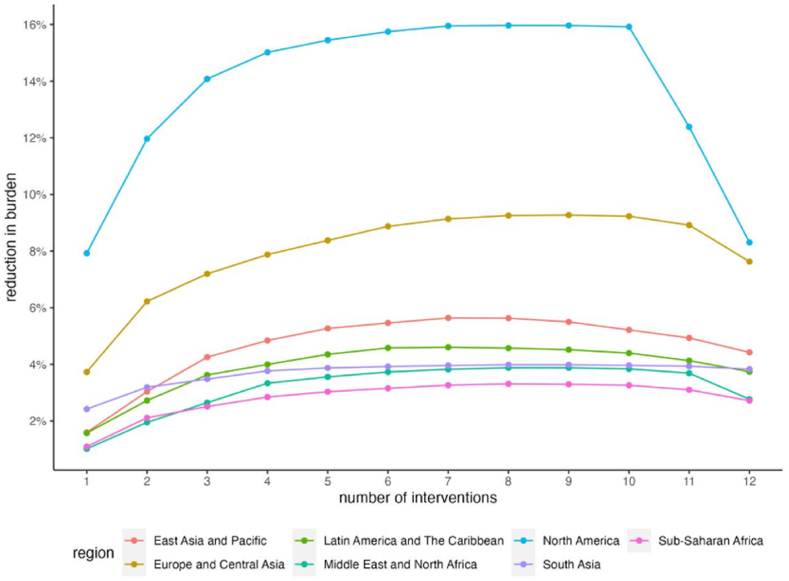
The proportion of disease burden reduction achieved by varying numbers of intervention components in the optimized decision-making packages.

Fig. 4 displays the proportion of disease burden reduction achieved by varying numbers of intervention components in the optimized decision-making packages. It was observed that, among the 4095 intervention packages formed by the 12 intervention measures, the optimal intervention combinations tend to include between 7 and 9 measures, as opposed to utilizing the full spectrum of available interventions. Furthermore, the efficacy of intervention strategies differs by regions, with North America and Europe and Central Asia experiencing more favorable outcomes.

Expanding on this, we focused on identifying optimal intervention combinations for different regions. Seven of the twelve interventions consistently appeared in the optimal intervention package for each region: raising low-carbon energy use, increasing energy intensity, improving livestock feed, expanding basic health care delivery coverage, enhancing health financing capacity, addressing air pollution, and improving road infrastructure construction. The optimal package for Europe and Central Asia also prioritized improvements in sanitation conditions and expanded pneumococcal vaccine coverage. The Middle East and North Africa's optimal package also incorporates expanded pneumococcal vaccine coverage. North America's package includes improvements in sanitation conditions, and forest conservation. South Asia's package focuses on expanded pneumococcal vaccine coverage. Sub-Saharan Africa's package includes expanded pneumococcal vaccine coverage. However, interventions like manure management and fertilizer do not frequently occur in the optimal combinations.

**Fig. 5:**
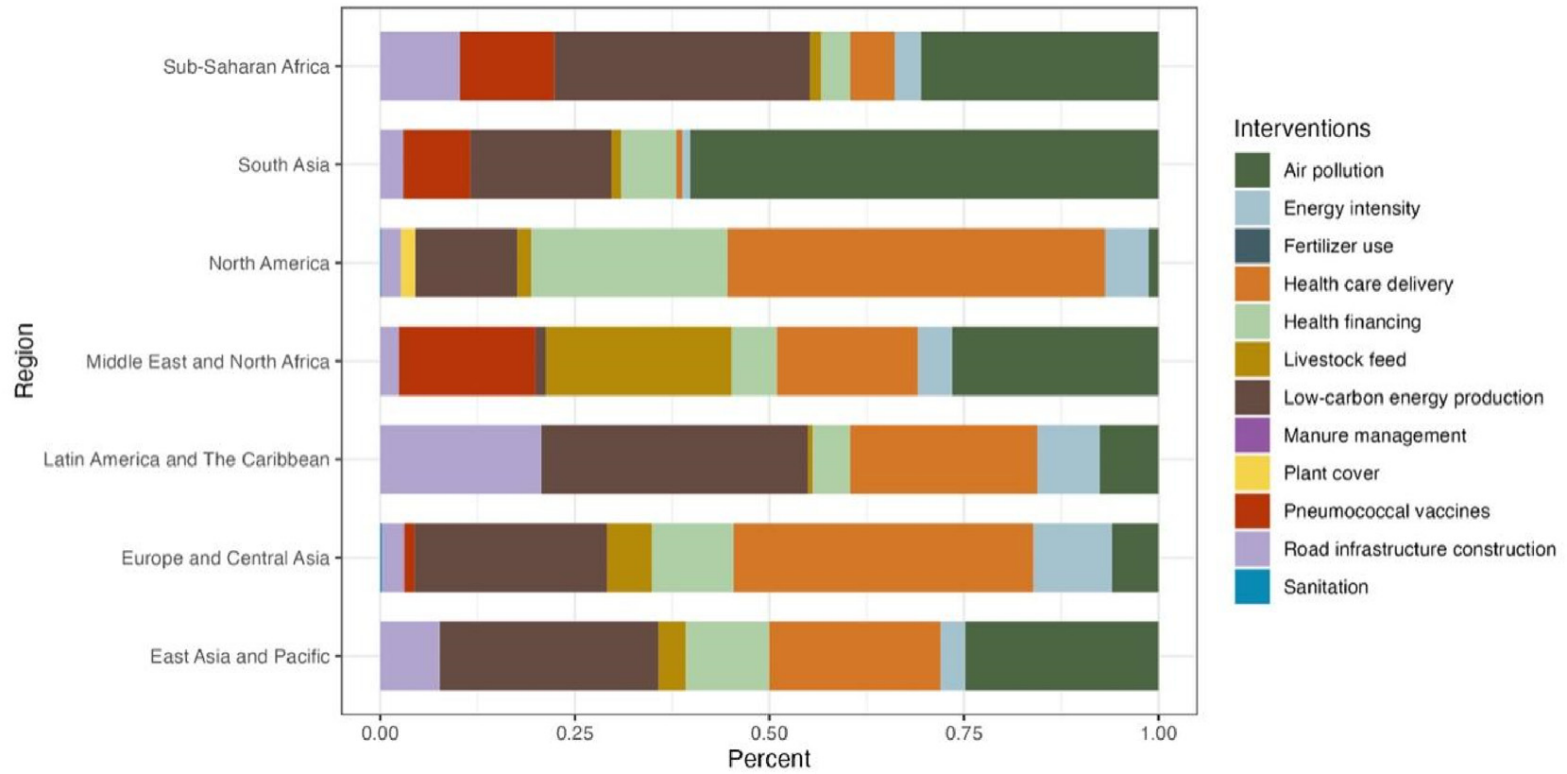
Breakdown of contributions of optimized decision-making packages to relieving HTD burden, per region.

Furthermore, we explored the contribution of different interventions within each region's optimized decision-making packages to the reduction in disease burden. Fig. 5 and Table 3 present the contributions of each intervention. Results indicate that raising low-carbon energy use is more effective for Latin America and The Caribbean, Sub-Saharan Africa, and East Asia and Pacific, with contributions of 34.26%, 32.87%, and 28.14% respectively. Air pollution control contributes to over 60% of disease burden reduction in South Asia and holds a significant share for the Middle East and North Africa (26.50%). For North America and Europe and Central Asia, the most effective strategy is expanding basic health service coverage, contributing 48.53% and 38.54% respectively.

**Table 3:** Breakdown of contributions of optimized decision-making packages to relieving HTD burden, per region.

	East Asia and Pacific	Europe and Central Asia	Latin America and The Caribbean	Middle East and North Africa	North America	South Asia	Sub-Saharan Africa
Air pollution	24.84%	5.94%	7.57%	26.50%	1.23%	60.18%	30.54%
Energy intensity	3.18%	10.13%	8.01%	4.42%	5.64%	1.03%	3.37%
Fertilizer use	/	/	/	/	/	/	/
Health care Delivery	22.02%	38.54%	24.07%	18.13%	48.53%	0.71%	5.71%
Health Financing	10.68%	10.45%	4.83%	5.79%	25.21%	7.12%	3.69%
Livestock feed	3.51%	5.78%	0.56%	23.88%	1.75%	1.26%	1.45%
Low-carbon energy production	28.14%	24.74%	34.26%	1.32%	13.12%	18.12%	32.87%
Manure Management	/	/	/	/	/	/	/
Plant cover	/	/	/	/	1.81%	/	/
Pneumococcal vaccines	/	1.31%	/	17.54%	/	8.58%	12.13%
Road Infrastructure construction	7.63%	2.92%	20.71%	2.42%	2.59%	2.99%	10.24%
Sanitation	/	0.18%	/	/	0.11%	/	/

#### 3.3.3 Sensitivity analysis

From intervention intensities ranging from 10% to 30%, the disease burden decreased by 6.41 in East Asia and Pacific, 2.50 in Europe and Central Asia, 3.17 in Latin America and The Caribbean, 7.89 in the Middle East and North Africa, 3.15 in North America, 9.17 in South Asia, and 7.04 in Sub-Saharan Africa. The results of the sensitivity analysis, detailed in **Appendix G**, confirm the robustness of our findings under various model parameters.

## 4 Discussion

This study constructs a comprehensive model designed to tackle the burden of HTDs through a systematic, multi-institutional, interdisciplinary, and cross-regional approach. Central to our framework is the integration of complex real-world factors into a predictive model that delineates the intricate interplay among variables critical in decision-making processes. By harnessing the One Health concept, our model not only simulates intervention strategies but also optimizes decision-making to mitigate the impacts of global warming on HTDs. We emphasize the cascading and complex nature of risks posed by global warming, highlighting spatial disparities and the sectoral impacts at both country and regional levels.

The diversity in optimal intervention packages points to the importance of recognizing each region's unique challenges and resources. Effective policies should be comprehensive and customized to the specific economic and environmental characteristics of each region. This is in line with the IPCC's Sixth Assessment Report, which suggests feasible, effective, and low-cost climate change mitigation and adaptation options, though they vary across different systems and regions (Lee et al., 2023). Our findings reveal the importance in East Asia and Pacific of addressing air quality issues and transitioning to sustainable energy. The region's diverse economic systems and climatic types are accompanied by severe environmental challenges. For instance, a study based in China emphasizes the significance of ambitious climate policies and low-carbon energy transitions, coupled with stringent clean-air policies, in mitigating health threats under climate change (Liu et al., 2022). In Europe and Central Asia, healthcare services and low-carbon energy production play key roles. The region's economic stability and robust welfare systems enable substantial investment in health infrastructure. The increasingly applied heat-health action plans (HHAPs) emphasize accurate and timely alert systems and the preparedness of the health and social care system (Martinez, Linares, et al., 2019). Meanwhile, the focus on energy intensity and low-carbon energy aligns with Europe's advanced energy policies and commitments to reducing greenhouse gas emissions, potentially mitigating the increasing frequency and intensity of heatwaves. According to a study analyzing over 80 solutions to climate change, a low-carbon society should be at the core of climate change policies (Mailloux et al., 2021). In Latin America and the Caribbean, besides optimizing healthcare services and low-carbon energy production, road infrastructure construction needs prioritization. Road safety issues are prominent in the region (Martinez et al., 2019), and ambient temperature affects road conditions, motor vehicles, and the physical and mental health of drivers. At the same time, per capita investment in traffic infrastructure is related to the burden of road injuries caused by high temperatures (He et al., 2023). In the Middle East and North Africa, the simulation results' emphasis on air pollution and livestock feed interventions highlights the region's struggle with environmental issues such as desertification and water scarcity. Livestock emissions account for about one-third of global anthropogenic methane emissions (Zhang et al., 2022). Improving feed quality/efficiency is a key strategy to increase livestock productivity and reduce emissions, although its long-term impact on human health warrants further exploration. In North America, the simulation results underscore the importance of healthcare services and health financing, indicating a strategy to leverage economic strength to enhance the health system's resilience to high temperatures. Cooling centers are increasingly used in major cities like Los Angeles, New York City, and Toronto, necessitating research and prioritizing medical resource allocation planning for high-risk subpopulations, applicable not only to cooling centers but also to emergency rooms and other facilities (Kim et al., 2021). As the climate continues to warm and moisten, medical institutions must address the increasing incidence of HTDs (Freifeld et al., 2023). In South Asia, the simulation results highlight a significant concern for air pollution. A World Bank report points out that 9 out of the 10 cities with the worst air pollution are in South Asia, leading to approximately 2 million premature deaths in the region annually (World Bank, 2022). Heatwaves and air pollution have a deadly impact on human health in South Asia; thus, urgent action is required to curb air pollution (Hasan et al., 2023). In sub-Saharan Africa, air pollution, low-carbon energy production, and pneumococcal vaccines are particularly prominent in simulation results. Expanding pneumococcal vaccination and controlling air pollution could have significant benefits in reducing the impact of HTDs. Much work has been done regarding the current state, challenges, and impacts of pneumococcal vaccination in these regions (Erhabor et al., 2021; Onwuchekwa et al., 2020; Soeters et al., 2019), and vaccination remains the most cost-effective public health intervention.

In addition, our optimal intervention combinations for different disease causes offer a reference framework for prioritizing disease prevention and treatment efforts. Firstly, the strategies for cardiovascular and metabolic diseases show a significant overlap, focusing on three main interventions: low-carbon energy production, enhancing health care delivery, and addressing air pollution. Notably, the impact of environmental air pollution on cardiovascular diseases has been increasingly documented (de Bont et al., 2022). Secondly, the strategy for infectious respiratory diseases highlights the critical role of pneumococcal vaccination, underscoring the consensus in public health on the importance of vaccines in disease prevention. Thirdly, in the context of injuries exacerbated by high temperatures, intervention strategies call for an expanded focus on road infrastructure. The risks of road injuries and drowning could also potentially be mitigated through increasing the availability of existing medical services. Fourthly, reforestation significantly contributes to alleviating the impact of heat on non-infectious respiratory diseases, which may also be linked to air quality. Related articles explore the causal relationship between exposure to pollutants and respiratory health, and also propose interventions such as urban afforestation (D'Antona et al., 2022). Finally, we were surprised to find that improving livestock feed in Europe and Central Asia might effectively alleviate infectious respiratory system diseases. This may point to the need for more research in sustainable livestock production, antimicrobial resistance, malnutrition and respiratory co-infections.

From the development of framework, design of impact pathways, and selection of indicators, to the choice of superior models for data-driven simulation and the output of optimal decision-making, we proposed a comprehensive research paradigm for systematic modeling and policy simulation. As we know, decision-makers often face fragmented information, especially for broader One Health-related topics (Sinclair, 2019). By integrating the existing achievements of the IPCC, WHO, and the GBD, we have developed a detailed framework. Utilizing comprehensive national-level data and adopting advanced data-driven models, we cover as many human-animal-environment interface risk factors as possible to predict HTD burden and also identify customized intervention strategies sensitive to regional differences. The introduction of multi-institutional impact pathways is particularly important as it bridges the gap between different stakeholders such as public health departments and environmental agencies, creating a collaborative environment crucial for the effective implementation of identified intervention measures. This interconnected framework facilitates the ability of policymakers and health practitioners to make informed decisions, thereby optimizing resource allocation and intervention outcomes. By providing a clear, evidence-based pathway for mitigating the impact of HTDs, the adaptability and scalability of this model allows its use across different geographical and economic contexts. Building on this, our simulation experiments compared the predictive simulation effects of eight data-driven algorithms, showing the potential of GNN models in policy simulation studies to provide more accurate and comprehensive decision support. This is an intuitive outcome since GNN models embody the structural information embedded in the data while other models do not fully utilize the hidden relationships. In general, GNN models are promising deep-learning models that can revolutionize the analysis of non-Euclidean data (Li et al., 2022). Years ago, studies by O'Neill et al. (O'Neill et al., 2009) emphasized that new computer-based decision tools would be able to locally estimate health impacts related to high temperatures and achieve potential added value by implementing a range of preventive strategies. Therefore, this inclusive paradigm based on the GNN model allows for the incorporation of fragmented information, thus deriving optimal decisions based on existing limited data. Meanwhile, there has been increasing attention on exploring causal modeling. The Climate and Health Risk Tool (CHaRT) has recently been developed, establishing a causal pathway linking high temperatures with composite outcomes of heat-related morbidity and mortality. This approach provides insights into risk factors in Washington state, as well as the prioritization of risk reduction intervention measures (Hess et al., 2023). In the current era of big data and machine learning, there is the potential to maximize the ideal application of decision tools by incorporating regional information related to heat-health associations, population characteristics, energy use, climate, and other factors. Simultaneously, re-examining the complex relationships among human, animal, and environmental health could facilitate tailored and cutting-edge intervention strategies.

There are several limitations that cannot be ignored. Firstly, the disease burden indicators from the GBD 2019 data used in this study are estimates with their own significant limitations (Burkart et al., 2021), and the effects of other infectious diseases such as malaria, as well as cancer, mental health, and more, need to be considered. Secondly, due to the absence of comprehensive data from all countries globally, we have not accounted for the impacts of various intervention policies such as air conditioning, heat warning systems, sustainable transportation, and health education. This omission could lead to incomplete coverage of the intervention package. Thirdly, the absence of relevant cost-effectiveness survey data means that the comparison of intervention decision outcomes in this study does not involve economic analysis. However, in real-world decision scenarios, the economic dimension is crucial. Fourthly, our research relies on data-driven simulation models, specifically the use of GNN for policy simulation and decision-making. While GNNs offer significant advantages in capturing the structural information within data, their effectiveness depends on the quality and comprehensiveness of the input data.

Building on the insights from our current research, the understanding and management of HTDs require extensive future investigation. The refinement and validation of predictive models are critical areas for future research. Our initial efforts used national-level data; however, by incorporating more detailed and real-time data, we believe we can significantly improve the accuracy and reliability of these models. Strategically combining data-driven and model-driven methodologies will address the interpretability challenges and the ‘black box’ phenomenon often associated with purely data-driven approaches. This combination not only increases transparency but also strengthens the basis for robust causal inference. Furthermore, economic assessments of selected intervention measures are essential. By evaluating the cost-effectiveness of these measures, we can conduct comparative analyses of their practicality and effectiveness in real-world scenarios. This step is vital for justifying the rational allocation of resources to these policy and practical interventions. Additionally, cross-regional comparative analysis is another important research direction. Detailed comparisons of intervention outcomes across different regions will highlight the targeted efficacy of various strategies, thereby deepening our understanding of how regional differences in climate, healthcare infrastructure, and socio-economic factors affect intervention success. Lastly, it is crucial to conduct longitudinal studies on the effects of intervention measures to monitor and evaluate the long-term efficacy of identified optimal intervention strategies in reducing the burden of HTDs.

## 5 Conclusion

In conclusion, our study represents a comprehensive and systematic effort in modeling and simulating decision-making in relieving global HTD burden based on the One Health approach. We have developed optimal intervention strategies tailored to the various disease causes and regions. The decision-making tool developed, which is grounded in systems thinking and the One Health concept, aims to aid different countries and regions in understanding specific heat-health connections and vulnerabilities in their locales. It also facilitates the implementation of evidence-based, optimal intervention strategies.

The escalating global warming crisis underscores the urgency of addressing HTDs, positioning it as a priority on public health agendas worldwide. Tackling this challenge necessitates a unified, interdisciplinary approach on a global scale. It is essential that we adopt a systematic approach, emphasizing context-specific evidence-based decision-making, to effectively confront this looming threat.

## Disclosure of potential conflicts of interest

The authors declare that they have no conflict of interest.

## Ethics approval for research involving human participants

This research study did not involve collection of data from human participants. Ethics approval is not required.

## Informed consent

Informed consent was not required since there were no individual participants in this study.

## Data and code availability

The data used in the calculations are publicly available and the sources are stated in the appendix. The full datasets and the codes are available, following manuscript publication, upon request from the corresponding author (Xiao-Xi Zhang, zhangxiaoxi@sjtu.edu.cn)

## Funding

The project was supported by 10.13039/501100001809National Natural Science Foundation of China (No. 72204160), the International Joint Laboratory on Tropical Diseases Control in Greater Mekong Subregion (No. 21410750200), and Open Research Project of Key Laboratory of Parasite Pathogen and Vector Biology of 10.13039/501100018684National Health Commission of the People's Republic of China (No. NHCKFKT2022-16).

## CRediT authorship contribution statement

**Xin-Chen Li:** Conceptualization, Data curation, Formal analysis, Methodology, Validation, Visualization, Writing – original draft, Writing – review & editing, Investigation, Project administration. **Hao-Ran Qian:** Conceptualization, Data curation, Formal analysis, Methodology, Project administration, Software, Validation, Visualization, Writing – review & editing, Investigation. **Yan-Yan Zhang:** Conceptualization, Data curation, Investigation, Methodology, Writing – review & editing. **Qi-Yu Zhang:** Data curation, Investigation, Methodology, Writing – review & editing. **Jing-Shu Liu:** Data curation, Writing – review & editing. **Hong-Yu Lai:** Methodology, Writing – review & editing. **Wei-Guo Zheng:** Writing – review & editing. **Jian Sun:** Writing – review & editing. **Bo Fu:** Methodology, Project administration, Supervision, Conceptualization, Resources, Software, Writing – review & editing. **Xiao-Nong Zhou:** Conceptualization, Funding acquisition, Methodology, Project administration, Resources, Supervision, Writing – review & editing. **Xiao-Xi Zhang:** Conceptualization, Formal analysis, Funding acquisition, Investigation, Methodology, Project administration, Resources, Supervision, Validation, Writing – original draft, Writing – review & editing.

## Declaration of competing interest

Xiao-Nong Zhou is on the editorial board of the journal Infectious Diseases Modeling. He was not involved in the peer-review or handling of the manuscript. The authors have no other competing interests to disclose.
