# Supplementary material for: Optimal decision-making in relieving global high temperature-related disease burden by data-driven simulation
Source: Infect Dis Model. 2024 Mar 19;9(2):618–33. doi: 10.1016/j.idm.2024.03.001 (PMC11026972; doi:10.1016/j.idm.2024.03.001)
Supplement: Multimedia component 1 [file mmc1.docx]

**Appendix A: Document Retrieval**

To develop a pathway of global warming impacts on high temperature-related diseases (HTDs), we conducted a systematic literature review. The review followed PRISMA guidelines(Liberati et al., 2009) and contains two main features: developing research questions and determining the search strategy. The following research questions were formulated for this review:

- RQ1. How does global warming (or high temperature or heat wave) affect HTDs? What causes of outcomes are likely to result?
- RQ2. What factors may exacerbate the risks of HTDs brought about by global warming, considered from the perspectives of exposure, risk, and vulnerability?
- RQ3. What interventions are available for HTDs, including climate change-related mitigation and adaptation measures?

To answer the above research questions, we developed the first set of keywords as shown in **Table A.1**.

Table A.1. The first set of keywords used in the present review.

| **Row** | **Set** |
| --- | --- |
| Test set 1 | (High temperature-related disease OR high temperature-related illness OR heat-related disease OR heat-related illness) |
| Test set 2 | (Global warming OR high temperature OR heat wave) |
| Search 1 | #1 AND #2 |
| Test set 3 | (Risk factors OR interventions) |
| Search 2 | #1 AND #3 |

Web of Science and Pub Med were used as database search tools in the present review. The search conducted in this review was divided into two steps: (a) the first set of keywords was used to discover relevant articles (633+1200 articles have been identified and added to the central database), and (b) a new set of keywords was developed based on the identified papers, which was used to discover additional articles. The second set of keywords is shown in **Table A.2**. The second set of keywords was developed to identify papers that did not mention specific keywords. Finally, 724+1356 articles with relevant content were identified.

Table A.2. The second set of keywords used in the present review.

| **Row** | **Set** |
| --- | --- |
| Test set 1 | (High temperature-related disease OR high temperature-related illness OR heat-related disease OR heat-related illness) |
| Test set 2 | (Hazard OR vulnerability OR exposure) |
| Search 1 | #1 AND #2 |
| Test set 3 | (Climate change OR global warming) |
| Test set 4 | (Mitigation OR adaptation) |
| Search 2 | #1 AND #3 AND #4 |
| Test set 5 | (Metabolic disease OR cardiovascular diseases OR non-infectious respiratory diseases OR infectious respiratory diseases OR injury) |
| Test set 6 | (Global warming OR high temperature OR heat wave) |
| Search 3 | #1 AND #5 AND #6 |

After the development of the main database and the identification of all relevant papers, a formal screening process was applied to the database based on the exclusion and inclusion criteria. The inclusion criteria were as follows:

1. Articles related to heat-related diseases;
2. Articles published from 2010 to 2023;
3. Articles related to research questions;
4. Articles written in English.

The exclusion criteria were as follows:

1. Papers written in other languages;
2. Articles from secondary sources that were not free or open access.

A chart of the selection strategy according to PRISMA guidelines is displayed in **Fig.A.1**. As a result, the present review included a total of 253 papers.


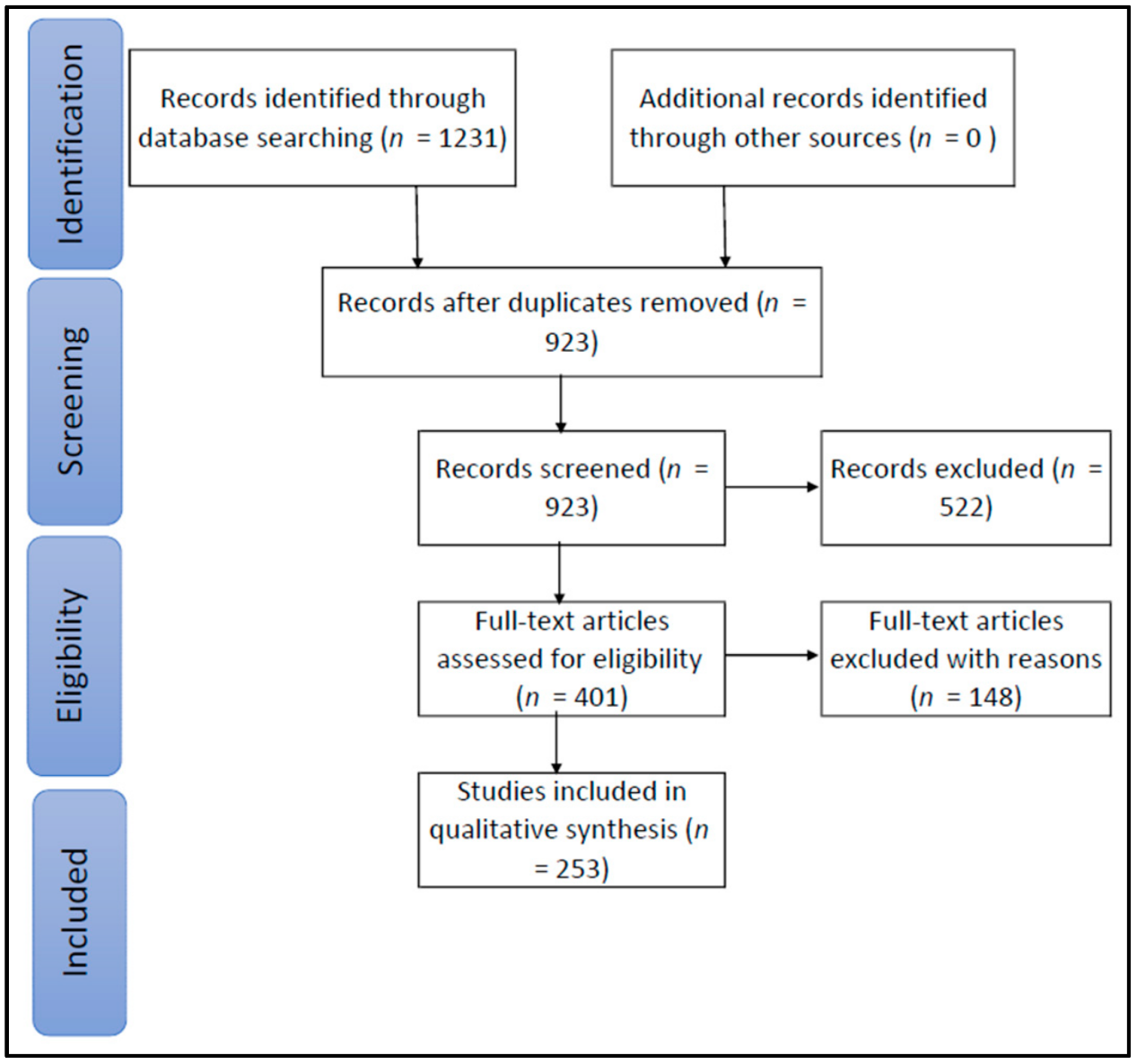


Fig.A.1. Chart of the selection strategy following PRISMA guidelines

**Reference:**

Liberati, A., Altman, D. G., Tetzlaff, J., Mulrow, C., Gøtzsche, P., & Ioannidis, J. (2009). The PRISMA statement for reporting systematic and meta‐analyses of studies that evaluate interventions: explanation and elaboration. *PLoS medicine*, *6*(7), 1-28.
