# Supplementary material for: Optimal decision-making in relieving global high temperature-related disease burden by data-driven simulation
Source: Infect Dis Model. 2024 Mar 19;9(2):618–33. doi: 10.1016/j.idm.2024.03.001 (PMC11026972; doi:10.1016/j.idm.2024.03.001)
Supplement: Multimedia component 2 [file mmc2.docx]

**Appendix B: Composition of the causes**

Table B.1. Classification and rate value of DALYs of high temperature-related diseases causes in 2019

| Causes Classification  in this paper | GBD Classification | Causes | DALYs per 100 000 |
| --- | --- | --- | --- |
| Metabolic Disease | Non-communicable disease | Diabetes mellitus | 10.12 |
|  |  | Chronic kidney disease | 2.86 |
| Cardiovascular Diseases |  | Ischemic heart disease | 12.59 |
|  |  | Stroke | 14.08 |
|  |  | Hypertensive heart disease | 0.46 |
| Non-infectious Respiratory Diseases |  | Chronic obstructive pulmonary disease | 3.84 |
| Infectious Respiratory Diseases | CMNND | Lower respiratory infections | 60.96 |
| Injury | Injury | Road injuries | 18.28 |
|  |  | Drowning | 8.59 |
|  |  | Exposure to mechanical forces | 1.55 |
|  |  | Self-harm | 9.34 |
|  |  | Interpersonal violence | 8.53 |


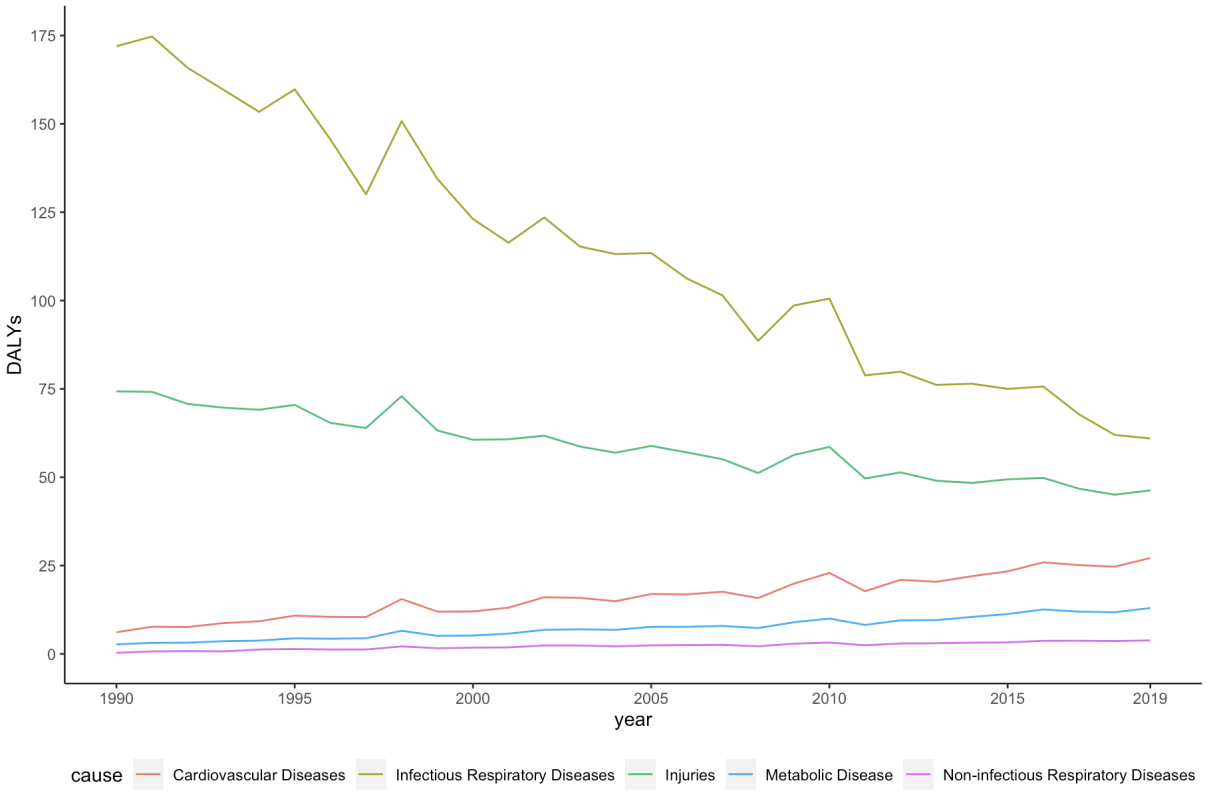


Fig.B.2. The trend of DALYs of different causes of high temperature-related diseases from 1990 to 2019
