# Supplementary material for: Optimal decision-making in relieving global high temperature-related disease burden by data-driven simulation
Source: Infect Dis Model. 2024 Mar 19;9(2):618–33. doi: 10.1016/j.idm.2024.03.001 (PMC11026972; doi:10.1016/j.idm.2024.03.001)
Supplement: Multimedia component 4 [file mmc4.pdf]

Appendix D

1 Models Screening

We initially built up a model pool for screening, which consists of 8 models designed using different methods. For each model, the input variable are variables from 2010 to 2019 excluding 5 intermediate outcome variables and 1 outcome variable y_{GBD} (i.e.high-temperature-related disease burden). In other words, the input variable are $\{x^{(n)}\}_{n=1}^{N} = \{x_{i,t}^{(n)}\}_{n=1}^{N}$ where (n) denotes n^{th} country, N denotes the number of samples in training dataset, $1 \leq i \leq 31$ denotes input variable indicator and t denotes time indicator from 2010 to 2019. Then, we use each model to predict HTD burden y_{GBD}^{n} in 2019.

In addition, we find that the range of data of each term varies greatly. For example, the data of the term, road infrastructure construction, shows the rank of road quality while the data of the term, metabolic disease, is the GBDs (global burden of disease) of metabolic disease due to high temperature and may varies from negative value to large positive value. To obtain better optimization of all models, we rescale the data of terms (including 31 input variables and 5 intermediate outcome variables) to [0, 1] respectively (i.e. min-max normalization). To be more specific, for input variables, we obtain the rescaled value by the following formula.

$$x_{i,t}^{\prime(n)} = \frac{x_{i,t}^{(n)} - \min\limits_{n',t'} x_{i,t'}^{(n')}}{\max\limits_{n',t'} x_{i,t'}^{(n')} - \min\limits_{n',t'} x_{i,t'}^{(n')}}$$

Then, we can derive intermediate outcome variables in the same way. For simplicity, in the subsequent formulas and equations, we use x and y instead of x' and y' respectively.

1.1 Model A: Linear regression

We employ the classic linear regression model which aims to establish a linear relationship between the input variables and outcome variable. The formula for linear regression can be expressed as follows.

$$\widehat{y}_{GBD} = \alpha + \sum_{i=1}^{31} \sum_{t=2010}^{2019} \beta_{i,t} x_{i,t} + \epsilon$$

where ϵ denotes the error term. The goal is to estimate the values of α and $\{\beta_{i,t}\}$ that minimize the sum of squared residuals (i.e., $\sum_{n=1}^{N}(y_{GBD}^{(n)} - \widehat{y}_{GBD}^{(n)})^2$).

1.2 Model B: Decision tree regression

We utilize Decision tree regression (Gordon et al. (1984)) to construct a tree-like structure to make predictions based on the input variables. To construct a tree-like structure, we start by selecting a splitting variable and a corresponding splitting point. Specifically, if we select $x_{i,t}$ as

splitting variable and splitting point as s, we obtain two areas including $R_1(i,t,s) = \{x|x_{i,t} \leq s\}$ and $R_2(i,t,s) = \{x|x_{i,t} > s\}$. To obtain optimal i,t and s, we need to solve the following equation.

$$\min_{i,t,s}\left[\min_{c_1}\sum_{x_{i,t}\in R_1(i,t,s)}(y_{GBD}^{(n)}-c_1)^2+\min_{c_2}\sum_{x_{i,t}\in R_2(i,t,s)}(y_{GBD}^{(n)}-c_2)^2\right]$$

where c_1 and c_2 denotes the centroids of two partitioned areas and are easy to derived by the following equations.

$$c_1 = \frac{1}{\left|\{x_{i,t}|x_{i,t}\in R_1(i,t,s)\}\right|}\sum_{x_{i,t}\in R_1(i,t,s)} y_{GBD}^{(n)}$$

$$c_2 = \frac{1}{\left|\{x_{i,t}|x_{i,t}\in R_2(i,t,s)\}\right|}\sum_{x_{i,t}\in R_2(i,t,s)} y_{GBD}^{(n)}$$

With the equations above, we can iteratively choose splitting variables and splitting points to obtain certain numbers of partitioned areas $R_1, R_2, ..., R_M$. Then, the decision tree regression can be expressed as the following formula.

$$\widehat{y}_{GBD} = \sum_{m=1}^{M} c_m I(x \in R_m)$$

1.3 Model C: Support vector regression (SVR)

SVR (Drucker et al. (1996)) aims to find a hyperplane that best fits the data in a high-dimensional feature space. Given input variables, it achieves the regression task by minimizing the difference between the predicted result and the actual result, while taking into account the maximum gap between the support vector and the hyperplane. To be more specific, given the hyperplane $f(x) = \sum_{i,t}\omega_{i,t}x_{i,t} + b$, we need to solve the following optimization problem.

$$\begin{aligned}\min_{\omega_{i,t},b,\xi^{(n)},\widehat{\xi}^{(n)}} \quad & \frac{1}{2}\sum_{i,t}\omega_{i,t}^2 + C\sum_{n=1}^{N}(\xi^{(n)}+\widehat{\xi}^{(n)})\\ \text{s.t.} \quad & f(x^{(n)}) - y_{GBD}^{(n)} \leq \epsilon + \xi^{(n)}\\ & y_{GBD}^{(n)} - f(x^{(n)}) \leq \epsilon + \widehat{\xi}^{(n)}\\ & \xi^{(n)}, \widehat{\xi}^{(n)} \geq 0\end{aligned}$$

where C denotes a penalty parameter while ϵ denotes a parameter that controls the width of the margin. After solving this optimization problem, we can predict the outcome variable by $f(x) = \sum_{i,t}\omega_{i,t}x_{i,t} + b$.

1.4 Model D: Adaboost

We employ Adaboost (Drucker (1997)) model which is an ensemble learning algorithm designed to enhance the accuracy of regression algorithms. It achieves this by training on weighted data in each round and adjusting weights in each round for model aggregation. Adaboost focuses each round of training on the error samples encountered during previous training rounds. The final model combines individual sub-models with weights to generate the ultimate predictive outcome. The procedure is as follows.

a. Set the initial weight vector w_1 such that $w_1^{(n)} = 1/N$ for $1 \leq n \leq N$.

b. Starting from $j = 1$, we can obtain a base learner h_j (e.g., decision tree regressor) with the training dataset and weight w_j.

c. Calculate the adjusted error $e_j^{(n)}$ for each instance by $E_j = \max_n |y_{GBD}^{(n)} - h_j(x^{(n)})|$ and $e_j^{(n)} = |y_{GBD}^{(n)} - h_j(x^{(n)})|/E_j$.

d. Calculate the adjusted error of h_j by $\epsilon_j = \sum_{n=1}^{N} e_j^{(n)} w_j^{(n)}$. If $\epsilon_j \geq 0.5$, go to step 7 and set $J = j$.

e. Let $\beta_j = \frac{\epsilon_j}{1-\epsilon_j}$

f. Update the weight vector $w_{j+1}^{(n)} = w_j^{(n)} \beta_j^{1-e_j^{(n)}} / Z_j$ and $Z_j = \sum_{n=1}^{N} w_j^{(n)} \beta_j^{1-e_j^{(n)}}$ denotes a normalizing constant. Then, go to step 2 with $j \leftarrow j + 1$.

g. Output the base learner (i.e. the weighted median of $h_j(x)$ for $1 \leq j \leq J$, using $\ln(1/\beta_j)$ as the weight for base learner $h_j(x)$.).

Then, we can predict the outcome variable with the base learner output by the steps above.

1.5 Model E: Random Forest (RF)

RF (Breiman (2001)) shares a similar concept with Adaboost and thus it is also an ensemble learning method that makes predictions by constructing multiple decision trees and combining their outputs. Each decision tree is trained on a different subset of the training data obtained through bootstrap sampling, and their predictions are aggregated using averaging techniques. Details are as follows.

a. Apply bootstrap sampling to generate multiple bootstrap samples from the original training dataset.

b. Select a subset of variables from the entire set of variables using a specific method or criteria.

c. Train a base learner (e.g., decision tree regressor) by utilizing the chosen samples and selected variables.

d. Repeat steps 1 to 3 for a specified number of iterations. Then, compute the average of all base learners to create the random forest model.

1.6 Model F: XGBoost

XGBoost (Chen & Guestrin (2016)) is an implementation of Gradient Boosting Trees, which improve predictive performance by iteratively training multiple decision trees within the boosting framework. XGBoost employs efficient optimization algorithms and regularization techniques, enabling it to handle large-scale datasets and high-dimensional features. The key steps of XGBoost are as follows.

a. Let $\widehat{y}_{GBD,0}^{(n)} = 0$ for $1 \leq n \leq N$.

b. Starting from $m = 1$, we hope to find a base learner f_m (e.g., decision tree regression) to fit $y_{GBD}^{(n)}$ with $\widehat{y}_{GBD,m}^{(n)} = \widehat{y}_{GBD,m-1}^{(n)} + f_m(x^{(n)})$.

c. Obtain the base learner f_m by minimizing $L_m + \Omega_m$. Here, $L_m = \sum_{n=1}^{(N)} (y_{GBD}^{(n)} - \widehat{y}_{GBD,m}^{(n)})^2$ and $\Omega_m = \lambda T + \frac{1}{2} \sum_{j=1}^{T} w_j^2$ where T is the number of leaf nodes in tree regression while w_j is the value of jth leaf node. Then, go to step 2 with $m \leftarrow m + 1$ until m equals a specified number of iterations (e.g., M).

d. The output of XGBoost is $\widehat{y}_{GBD,M}^{(n)} = \sum_{m=1}^{M} f_m(x^{(n)})$.

1.7 Model G: XGBoost Random Forest

It is an extension of the XGBoost algorithm that combines the concepts of Random Forest with XGBoost, creating a powerful ensemble learning model. It introduces randomness in the construction process of each decision tree, increasing the model's diversity and further enhancing its predictive capabilities. In fact, XGBoost random forest has similar steps to XGBoost. Nevertheless, in step 3, XGBoost random forest constructs a tree regressor f_m with bootstrap samples and a subset of variables.

1.8 Model H: Graph Neural Network (GNN)

In GNN model, we first employ LSTM to capture the intrinsic sequential feature and then utilize Graph Attention Network Module (GAT) to figure out the structural relationship. At last, we design an explainability loss module to make its prediction much more explainable. In summary, we predict GBD by the following formula.

$$\widehat{y}_{GBD}^{(n)} = \text{GAT}(\text{LSTM}(x^{(n)}))$$

For better illustration, we introduce our proposed GNN model by presenting important terminologies and show the detail of model in order.

1.8.1 Preliminary

Let $G = (V, E)$ denote a directed graph, where V and E represent the sets of nodes and directed edges, respectively. In our scenario, we have $|V|$ =37 nodes and $|E|$ =72 edges for each country or each graph.

We set an initial feature $\boldsymbol{x_i} \in \mathbb{R}^{10}$ for each node $v_i \in V$ in graph G, where $\boldsymbol{x_i}$ stands for ten years of data of this node. If we concatenate feature of all nodes, we obtain a matrix $X = [\boldsymbol{x_1},\ \boldsymbol{x_2},\ ...,\ \boldsymbol{x_{|V|}}]^T \in \mathbb{R}^{|V|\times 10}$ where $|V|$ is number of nodes in graph. We can interpret this matrix X in two ways. The first is that we have ten-year data $\boldsymbol{x_i} \in \mathbb{R}^{10}$ for each node, and we have $|V|$ nodes. The second is that we have $|V|-$node data $\boldsymbol{z_t} \in \mathbb{R}^{|V|}$ for each year from 2010 to 2019. If we concatenate them, we can also obtain a matrix $Z = [\boldsymbol{z_1},\ \boldsymbol{z_2},\ ...,\ \boldsymbol{z_{10}}]^T = X^T \in \mathbb{R}^{10\times|V|}$

1.8.2 Model Design

Time Series Processing Module

Since our data is temporal, we hope to capture the intrinsic feature in our ten-year data. Then we turn to a popular model LSTM (Hochreiter & Schmidhuber (1997); Graves & Graves (2012)) for better feature extraction. The idea behind the model has been well described in previous paper (Hochreiter & Schmidhuber (1997); Graves & Graves (2012)), so we focus on the process of how we obtain the hidden feature for each year.

In LSTM model,we first compute forget gate, input gate and output gate for each $\boldsymbol{z_t}$ by the following formulas.

$$\boldsymbol{f_t} = \sigma(U_f \boldsymbol{h_{t-1}} + W_f \boldsymbol{z_t} + \boldsymbol{b_f}), \quad \boldsymbol{i_t} = \sigma(U_i \boldsymbol{h_{t-1}} + W_i \boldsymbol{z_t} + \boldsymbol{b_i}), \quad \boldsymbol{o_t} = \sigma(U_o \boldsymbol{h_{t-1}} + W_o \boldsymbol{z_t} + \boldsymbol{b_o})$$

where $U_f,\ U_i,\ U_o,\ W_f,\ W_i,\ W_o \in \mathbb{R}^{|V|\times|V|},\ \boldsymbol{b_f},\ \boldsymbol{b_i},\ \boldsymbol{b_o} \in \mathbb{R}^{|V|}$ are trainable parameters in LSTM model.

With these three gate vector, we can derive the hidden feature $\boldsymbol{h_t}$ of each year by the following formulas.

$$\widetilde{C_t} = \tanh(U_C \boldsymbol{h_{t-1}} + W_C \boldsymbol{z_t} + \boldsymbol{b_C}), \quad C_t = \boldsymbol{f_t} \odot C_{t-1} + \boldsymbol{i_t} \odot \widetilde{C_t}, \quad \boldsymbol{h_t} = \boldsymbol{o_t} \odot \tanh(C_t)$$

where $U_C,\ W_C \in \mathbb{R}^{|V|\times|V|},\ \boldsymbol{b_C} \in \mathbb{R}^{|V|}$ are also trainable parameters.

With the calculation above, we have hidden feature $\boldsymbol{h_t} \in \mathbb{R}^{|V|}$ for ten years. Similarly, we obtain a matrix $H = [\boldsymbol{h_1},\ \boldsymbol{h_2},\ ...,\ \boldsymbol{h_{10}}]^T = \overline{X}^T \in \mathbb{R}^{10\times|V|}$.

Graph Attention Network Module

Besides temporal feature, we also hope to figure out the structural relationship behind the model. Since our data is modelled as a graph, it is natural for us to utilize GNN. To capture the internal correlation among different nodes, we turn to the well-known attention model, Graph Attention Network (GAT) (Veličković et al. (2017)).

Note that $\overline{X} = [\overline{\boldsymbol{x}}_{\boldsymbol{1}},\ \overline{\boldsymbol{x}}_{\boldsymbol{2}},\ ...,\ \overline{\boldsymbol{x}}_{\boldsymbol{|V|}}]^T = H^T \in \mathbb{R}^{|V|\times 10}$. Then, for node v_i in graph G, we update its feature as $\overline{\boldsymbol{x}}_{\boldsymbol{i}}$.

The key idea of GAT is that for every node, it first computes the attention scores between the node and its neighbors and then update its own feature by the normalized attention scores and neighbors' feature. The formulas are as follows.

$$
\begin{aligned}
\overline{\boldsymbol{z}}_{\boldsymbol{i}} &= W_{att}\overline{\boldsymbol{x}}_{\boldsymbol{i}} && \text{for } \forall v_i \in V \\
e_{ji} &= \text{LeakyReLU}(\boldsymbol{a^T}[\boldsymbol{z_j}||\boldsymbol{z_i}]) && \text{for } \forall (v_j, v_i) \in E \\
\alpha_{ji} &= \frac{\exp(e_{ji})}{\sum_{k\in\mathcal{N}(i)} \exp(e_{ki})} && \text{for } \forall (v_j, v_i) \in E \\
\overline{\boldsymbol{h}}_{\boldsymbol{i}} &= \sum_{j\in\mathcal{N}(i)} \alpha_{ji}\overline{\boldsymbol{z}}_{\boldsymbol{j}} && \text{for } \forall v_i \in V
\end{aligned}
$$

where $W_{att} \in \mathbb{R}^{10\times l_{att}}$ and $\boldsymbol{a} \in \mathbb{R}^{2l_{att}}$ denote trainable parameters and l_{att} denotes a hyper parameter. Note that $||$ stands for vector concatenation.

To enhance feature extraction, we introduce multi-head attention to our model. That is, if we replicate this process with different parameter $W_{att}^k,\ \boldsymbol{a^k},\ k \in \{1,\ 2,\ ...,\ K\}$, we will obtain $\overline{\boldsymbol{h}}_{\boldsymbol{i}}^{\boldsymbol{1}},\ \overline{\boldsymbol{h}}_{\boldsymbol{i}}^{\boldsymbol{2}},\ ...,\ \overline{\boldsymbol{h}}_{\boldsymbol{i}}^{\boldsymbol{K}}$ where K is also a hyper parameter. Then, for every node, we concatenate their hidden feature from different head to obtain its final feature $\widetilde{\boldsymbol{h}}_{\boldsymbol{i}} = [\overline{\boldsymbol{h}}_{\boldsymbol{i}}^{\boldsymbol{1}}||\overline{\boldsymbol{h}}_{\boldsymbol{i}}^{\boldsymbol{2}}||...||\overline{\boldsymbol{h}}_{\boldsymbol{i}}^{\boldsymbol{K}}] = ||_{k=1}^{K}\ \overline{\boldsymbol{h}}_{\boldsymbol{i}}^{\boldsymbol{k}} \in \mathbb{R}^{Kl_{att}}$.

Explainability Loss Module

In most predicting task, many scholars may define the loss as the Mean Square Error (MSE) between true value y_{GBD} and $\widehat{y}_{GBD} = W_y\widetilde{\boldsymbol{h}}_{\boldsymbol{y}} + b_y$. To be more specific, given predicted value $\widehat{y}_{GBD}$, we define

$$l_{tot} \triangleq (y_{GBD} - \widehat{y}_{GBD})^2.$$

We want to design a loss to help GNN model predict accurately.

First, we hope to fully utilize priori knowledge. Since we know that $y_{GBD} = \sum_{i=0}^{5} y_{GBD_i}$, we also hope that we can first predict five GBDs through our model, and then predict total GBD y by the sum of predicted values. Then, we can define the loss of five GBDs as

$$l_{GBD} \triangleq \sum_{i=1}^{5}(y_{GBD_i} - \widehat{y}_{GBD_i})^2.$$

Furthermore, we also hope to figure out the internal mechanism to improve the explainability of our model so that it can recover the value of certain nodes $\overline{V} = \{v_{m_1},\ v_{m_2},\ ...\}$ such as greenhouse gas emissions, distribution of air pollutants and so on. Then, we define the recovery loss as

$$l_{rec} \triangleq \sum_{v\in\overline{V}}(y_v - \widehat{y}_v)^2.$$

Finally, we can define the loss of our model

$$l = l_{tot} + \lambda_1 l_{GBD} + \lambda_2 l_{rec}$$

where λ_1 and λ_2 are hyper parameters.

The remaining question is how to obtain $\widehat{y}_{GBD_i}$ and $\widehat{y}_v$ by $\widetilde{\boldsymbol{h}}_{\boldsymbol{i}}$. We hope to fully utilize the information of nodes that we are interested in, and thus we apply a fully connected layer to concatenated feature of those nodes by the following formula.

$$[\widehat{y}_{GBD_1},\ ...,\ \widehat{y}_{GBD_5},\ y_{v_{m_1}},\ y_{v_{m_2}},\ ...] = W[\widetilde{\boldsymbol{h}}_{\boldsymbol{GBD_1}},\ ...,\ \widetilde{\boldsymbol{h}}_{\boldsymbol{GBD_5}},\ \widetilde{\boldsymbol{h}}_{\boldsymbol{v_{m_1}}},\ \widetilde{\boldsymbol{h}}_{\boldsymbol{v_{m_2}}},\ ...] + \boldsymbol{b}$$

where W and $\boldsymbol{b}$ are trainable parameters. In addition, the predicted total GBD is $\widehat{y}_{GBD_i} \triangleq \sum_{i=1}^{5} \widehat{y}_{GBD_i}$.

1.8.3 Setting

Hyper Parameter Setting

There are several hyper parameters including $l_{att},\ K,\ \lambda_1,\ \lambda_2$.

In attention module, we let $l_{att} = 5$ and $K = 10$ so that $\widetilde{\boldsymbol{h}}_{\boldsymbol{i}}$ can capture the hidden feature well.

In Explainability Module, since the total GBD is the sum of five GBDs, we let $\lambda_1 = 5$ in order to keep l_{GBD} as important as l_{tot}. Similarly, as for λ_2, we let $\lambda_2 = 1$ to keep l_{rec} as important as l_{tot}.

Loss Term Setting

Intuitively, we hope to recover the value of all nodes so that our model can learn a better hidden feature for each node. However, it will lead to the increase of trainable parameters greatly, and thus we focus on some significant nodes.

First, we find that some nodes do not have in-neighbour, which means that their hidden feature will not be updated in GAT module. Consequently, their hidden feature keep unchanged after the GAT module, and thus its representation contain less information. Then, recovering the value of these nodes will be unnecessary and difficult. As a result, we no longer focus on those nodes without in-neighbour.Similarly, nodes with few in-neighbour should also be excluded.

Therefore, the remaining nodes are those nodes that aggregate much information from in-neighbour. The remaining nodes include *low-carbon energy use, temperature change, distribution of air pollutants, greenhouse gas emissions from energy sector and greenhouse gas emissions from agricultural sector.*

2 Evaluation of model performance

We measure the goodness of model in three aspects. In the following equations, $y_{GBD}^{(n)}$ denotes the true GBD value of the nth country in 2019, $\widehat{y}_{GBD}^{(n)}$ denotes the predicted GBD value of the nth country in 2019 and $\overline{y}_{GBD}$ denotes the average of true GBD value in 2019.

First, we measure the actual error between the predicted value $\widehat{y}_{GBD}^{(n)}$ and the true value $y_{GBD}^{(n)}$. There are two common metrics to measure including Root Mean Squared Error (RMSE) and Mean Absolute Error (MAE). The smaller the RMSE or MAE, the more accurate the prediction. In addition, we also include Mean Squared Error (MSE) and Median Absolute Error (MedAE) to see models' performance in different metrics.

$$\text{RMSE} = \sqrt{\frac{1}{N}\sum_{n=1}^{N}\left(y_{GBD}^{(n)} - \widehat{y}_{GBD}^{(n)}\right)^2}$$

$$\text{MAE} = \frac{1}{N}\sum_{n=1}^{N}\left|y_{GBD}^{(n)} - \widehat{y}_{GBD}^{(n)}\right|$$

$$\text{MSE} = \frac{1}{N}\sum_{n=1}^{N}\left(y_{GBD}^{(n)} - \widehat{y}_{GBD}^{(n)}\right)^2$$

$$\text{MedAE} = median\left(\left|y_{GBD}^{(1)} - \widehat{y}_{GBD}^{(1)}\right|, \left|y_{GBD}^{(2)} - \widehat{y}_{GBD}^{(2)}\right|, \ldots, \left|y_{GBD}^{(n)} - \widehat{y}_{GBD}^{(n)}\right|\right)$$

Next, we measure the relative error between the predicted value $\widehat{y}_{GBD}^{(n)}$ and the true value $y_{GBD}^{(n)}$. We utilize Mean Absolute Percentage Error (MAPE) and Root Mean Squared Percentage Error (RMSPE) to measure. The closer the MAPE or RMSPE is to 1, the smaller the relative error.

$$\text{MAPE} = \frac{1}{N}\sum_{n=1}^{N}\left|\frac{y_{GBD}^{(n)} - \widehat{y}_{GBD}^{(n)}}{y_{GBD}^{(n)}}\right| * 100\%$$

$$\text{RMSPE} = \sqrt{\frac{1}{N}\sum_{n=1}^{N}\left(\frac{y_{GBD}^{(n)} - \widehat{y}_{GBD}^{(n)}}{y_{GBD}^{(n)}}\right)^2} * 100\%$$

As for the last aspect, we measure the correlation between the predicted value $\widehat{y}_{GBD}^{(n)}$ and the true value $y_{GBD}^{(n)}$. We utilize coefficient of determination (R2) to measure. The closer the R2 is to 1, the better the model fits.

$$R^2 = 1 - \frac{\sum_{n=1}^{N}\left(y_{GBD}^{(n)} - \widehat{y}_{GBD}^{(n)}\right)^2}{\sum_{n=1}^{N}\left(y_{GBD}^{(n)} - \overline{y}_{GBD}\right)^2}$$

If a model is equipped with small actual and relative errors and a R^2 close to 1, we say that this model can reflect the relationship between input factors and GBD values very well. Consequently, we hope to find a good model that performs well in these aspects.

References

Breiman, L. (2001). Random forests. *Machine learning*, *45*, 5–32.

Chen, T., & Guestrin, C. (2016). Xgboost: A scalable tree boosting system. In *Proceedings of the 22nd acm sigkdd international conference on knowledge discovery and data mining* (pp. 785–794).

Drucker, H. (1997). Improving regressors using boosting techniques. In *Icml* (Vol. 97, pp. 107–115).

Drucker, H., Burges, C. J., Kaufman, L., Smola, A., & Vapnik, V. (1996). Support vector regression machines. *Advances in neural information processing systems*, *9*.

Gordon, A., Breiman, L., Friedman, J., Olshen, R., & Stone, C. J. (1984). Classification and regression trees. *Biometrics*, *40*(3), 874.

Graves, A., & Graves, A. (2012). Long short-term memory. *Supervised sequence labelling with recurrent neural networks*, 37–45.

Hochreiter, S., & Schmidhuber, J. (1997). Long short-term memory. *Neural computation*, *9*(8), 1735–1780.

Veličković, P., Cucurull, G., Casanova, A., Romero, A., Lio, P., & Bengio, Y. (2017). Graph attention networks. *arXiv preprint arXiv:1710.10903*.
