# Supplementary material for: Optimal decision-making in relieving global high temperature-related disease burden by data-driven simulation
Source: Infect Dis Model. 2024 Mar 19;9(2):618–33. doi: 10.1016/j.idm.2024.03.001 (PMC11026972; doi:10.1016/j.idm.2024.03.001)
Supplement: Multimedia component 5 [file mmc5.docx]

**Appendix E: Model Simulation Result**

Table E.1. The comparison of the eight models

| **Model** | **RMSE** | **MSE** | **MAE** | **MedAE** | **MAPE** | **RMSPE** | **R2** |
| --- | --- | --- | --- | --- | --- | --- | --- |
| Linear Regression | 0.834909244 | 0.697073446 | 0.611833062 | 0.470664129 | 0.656222884 | 0.848224816 | -0.860087483 |
| Decision Tree Regression | 0.641157339 | 0.411082733 | 0.421886626 | 0.271792734 | 0.344875546 | 0.485118828 | -0.096943015 |
| Support Vector Regression | 0.569913359 | 0.324801237 | 0.30551538 | **0.09807745** | 0.215932409 | 0.289003232 | 0.133292597 |
| Adaboost | 0.491776082 | 0.241843715 | 0.318333291 | 0.199617674 | 0.250364111 | 0.323800674 | 0.354658437 |
| Random Forest | 0.497766523 | 0.247771511 | 0.320616266 | 0.19034152 | 0.264759576 | 0.348315948 | 0.338840564 |
| XGBoost | 0.619313096 | 0.383548711 | 0.395255545 | 0.18257425 | 0.323086451 | 0.502348965 | -0.023470571 |
| XGBoost Random Forest | 0.558741969 | 0.312192588 | 0.352762193 | 0.152954821 | 0.285247129 | 0.421213928 | 0.166937819 |
| **GNN** | **0.37064491** | **0.137377649** | **0.244706735** | 0.102603375 | **0.199298711** | **0.276374035** | **0.63341819** |


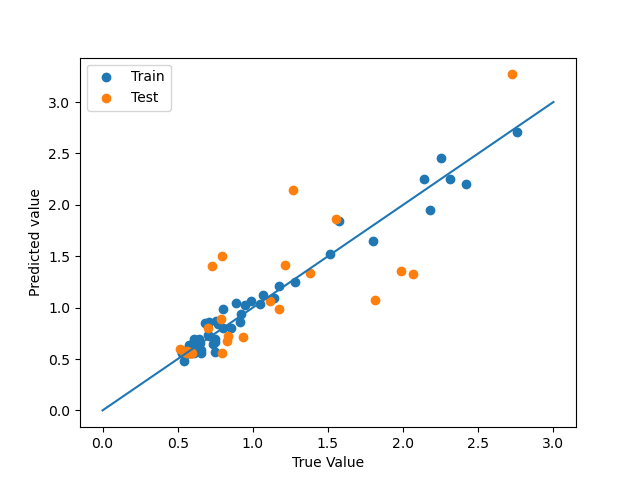


Fig.E.1. The fitting results of GNN model

As shown in Table E.1. and Fig.E.1., in terms of different metrics including actual and relative errors, GNN model shows better simulation performance. To be more specific, compared to other models, GNN model produces predicted values that are both actually closer and relatively closer to the true values.
