# Supplementary material for: Optimal decision-making in relieving global high temperature-related disease burden by data-driven simulation
Source: Infect Dis Model. 2024 Mar 19;9(2):618–33. doi: 10.1016/j.idm.2024.03.001 (PMC11026972; doi:10.1016/j.idm.2024.03.001)
Supplement: Multimedia component 6 [file mmc6.docx]

**Appendix F: Optimal Decision-making for Five Categories of Disease Causes**

1. Cardiovascular Diseases


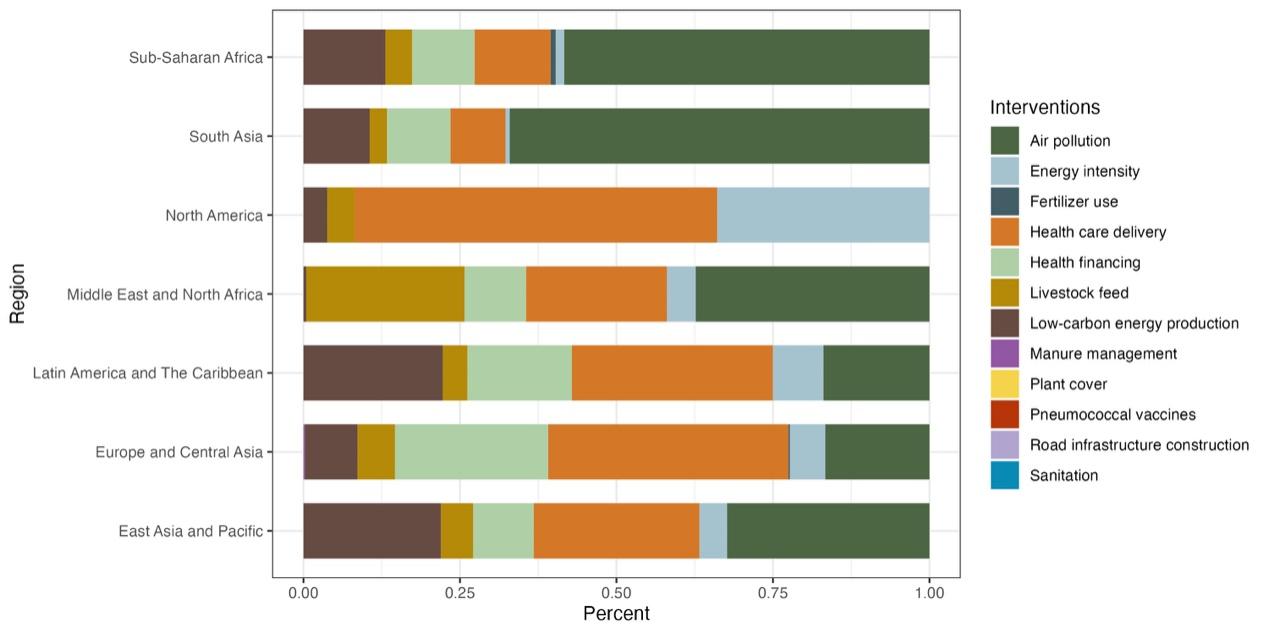


Fig.F.1. Breakdown of contributions of optimized decision-making packages to relieving HTD burden of cardiovascular diseases by regions.

Table F.1. Breakdown of contributions of optimized decision-making packages to relieving HTD burden of cardiovascular diseases by regions.

|  | East Asia and Pacific | Europe and Central Asia | Latin America and The Caribbean | Middle East and North Africa | North America | South Asia | Sub-Saharan Africa |
| --- | --- | --- | --- | --- | --- | --- | --- |
| Air pollution | 32.32% | 16.64% | 16.94% | 37.34% | / | 67.05% | 58.34% |
| Energy intensity | 4.43% | 5.65% | 8.08% | 4.60% | 33.91% | 0.68% | 1.37% |
| Fertilizer use | / | 0.22% | / | / | 0.01% | / | 0.80% |
| Health care Delivery | 26.49% | 38.40% | 32.11% | 22.52% | 57.93% | 8.78% | 12.18% |
| Health Financing | 9.65% | 24.46% | 16.69% | 9.78% | / | 10.12% | 9.94% |
| Livestock feed | 5.14% | 5.99% | 3.92% | 25.31% | 4.33% | 2.79% | 4.28% |
| Low-carbon energy production | 21.98% | 8.42% | 22.27% | 0.45% | 3.81% | 10.58% | 13.09% |
| Manure Management | / | 0.21% | / | / | / | / | / |
| Plant cover | / | / | / | / | / | / | / |
| Pneumococcal vaccines | / | / | / | / | / | / | / |
| Road Infrastructure construction | / | / | / | / | / | / | / |
| Sanitation | / | / | / | / | / | / | / |

1. Infectious Respiratory Diseases


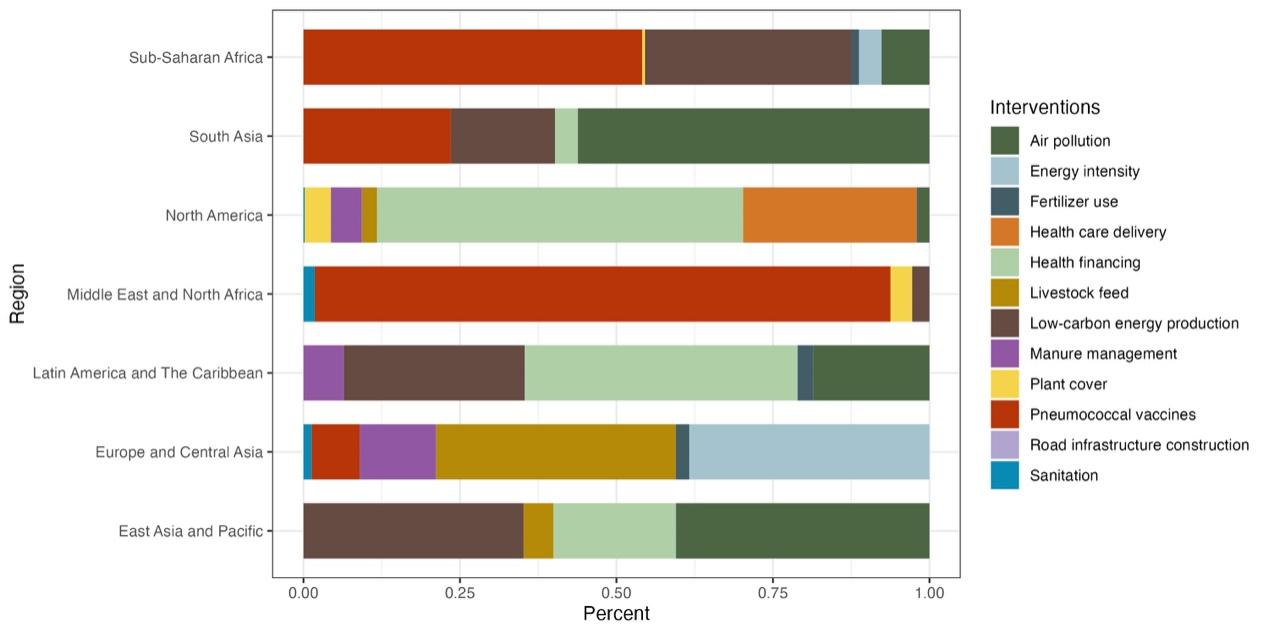


Fig.F.2. Breakdown of contributions of optimized decision-making packages to relieving HTD burden of infectious respiratory diseases by regions.

Table F.2. Breakdown of contributions of optimized decision-making packages to relieving HTD burden of infectious respiratory diseases by regions.

|  | East Asia and Pacific | Europe and Central Asia | Latin America and The Caribbean | Middle East and North Africa | North America | South Asia | Sub-Saharan Africa |
| --- | --- | --- | --- | --- | --- | --- | --- |
| Air pollution | 40.47% | / | 18.58% | / | 1.80% | 56.16% | 7.66% |
| Energy intensity | / | 38.36% | / | / | / | / | 3.61% |
| Fertilizer use | 0.05% | 2.16% | 2.48% | / | 0.24% | / | 1.26% |
| Health care Delivery | / | / | / | / | 27.76% | / | / |
| Health Financing | 19.55% | / | 43.59% | / | 58.45% | 3.64% | / |
| Livestock feed | 4.77% | 38.31% | / | / | 2.43% | / | / |
| Low-carbon energy production | 35.16% | / | 28.92% | 2.78% | / | 16.69% | 32.92% |
| Manure Management | / | 12.18% | 6.42% | / | 4.94% | / | / |
| Plant cover | / | / | / | 3.46% | 4.19% | / | 0.44% |
| Pneumococcal vaccines | / | 7.69% | / | 91.98% | / | 23.50% | 54.10% |
| Road Infrastructure construction | / | / | / | / | / | / | / |
| Sanitation | / | 1.30% | / | 1.79% | 0.19% | / | / |

1. Injuries


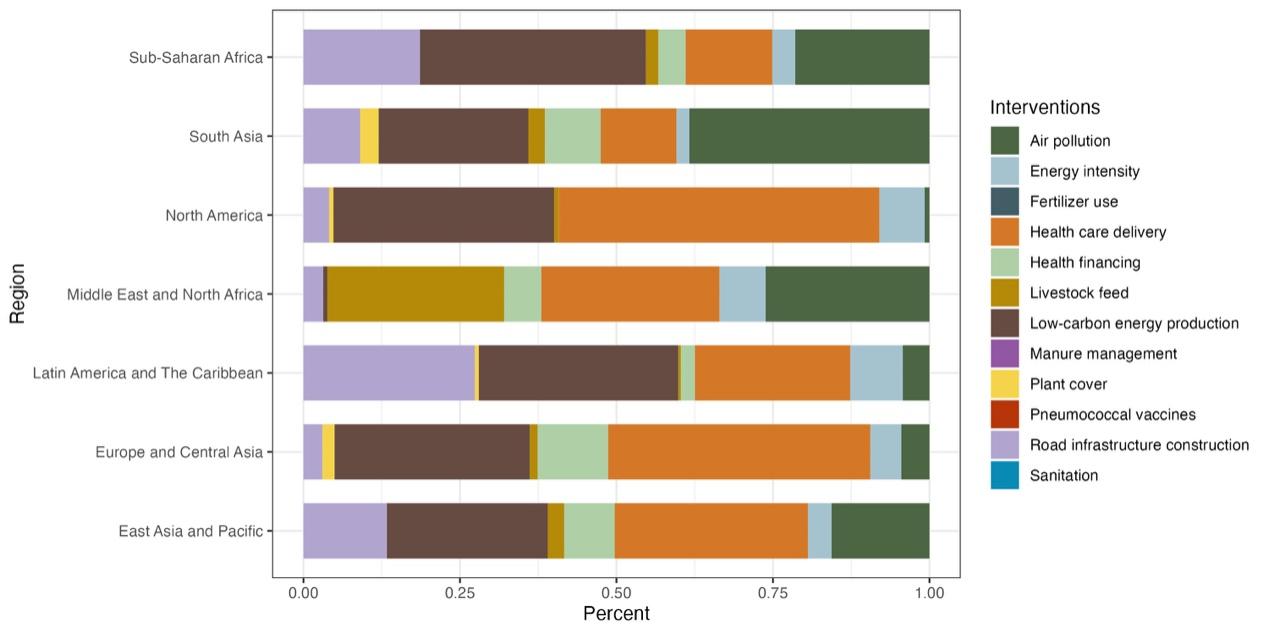


Fig.F.3. Breakdown of contributions of optimized decision-making packages to relieving HTD burden of injuries by regions.

Table F.3. Breakdown of contributions of optimized decision-making packages to relieving HTD burden of injuries by regions.

|  | East Asia and Pacific | Europe and Central Asia | Latin America and The Caribbean | Middle East and North Africa | North America | South Asia | Sub-Saharan Africa |
| --- | --- | --- | --- | --- | --- | --- | --- |
| Air pollution | 15.64% | 4.49% | 4.27% | 26.18% | 0.77% | 38.34% | 21.44% |
| Energy intensity | 3.77% | 4.97% | 8.39% | 7.37% | 7.24% | 2.09% | 3.66% |
| Fertilizer use | / | / | / | / | / | / | / |
| Health care Delivery | 30.88% | 41.88% | 24.82% | 28.43% | 51.24% | 12.10% | 13.87% |
| Health Financing | 8.06% | 11.24% | 2.23% | 5.95% | / | 8.88% | 4.33% |
| Livestock feed | 2.62% | 1.29% | 0.41% | 28.24% | 0.69% | 2.66% | 1.96% |
| Low-carbon energy production | 25.72% | 31.17% | 31.89% | 0.70% | 35.28% | 23.91% | 36.13% |
| Manure Management | / | / | / | / | / | / | / |
| Plant cover | / | 1.91% | 0.61% | / | 0.69% | 2.93% | / |
| Pneumococcal vaccines | / | / | / | / | / | / | / |
| Road Infrastructure construction | 13.31% | 3.05% | 27.39% | 3.12% | 4.09% | 9.08% | 18.61% |
| Sanitation | / | / | / | / | / | / | / |

1. Metabolic Diseases


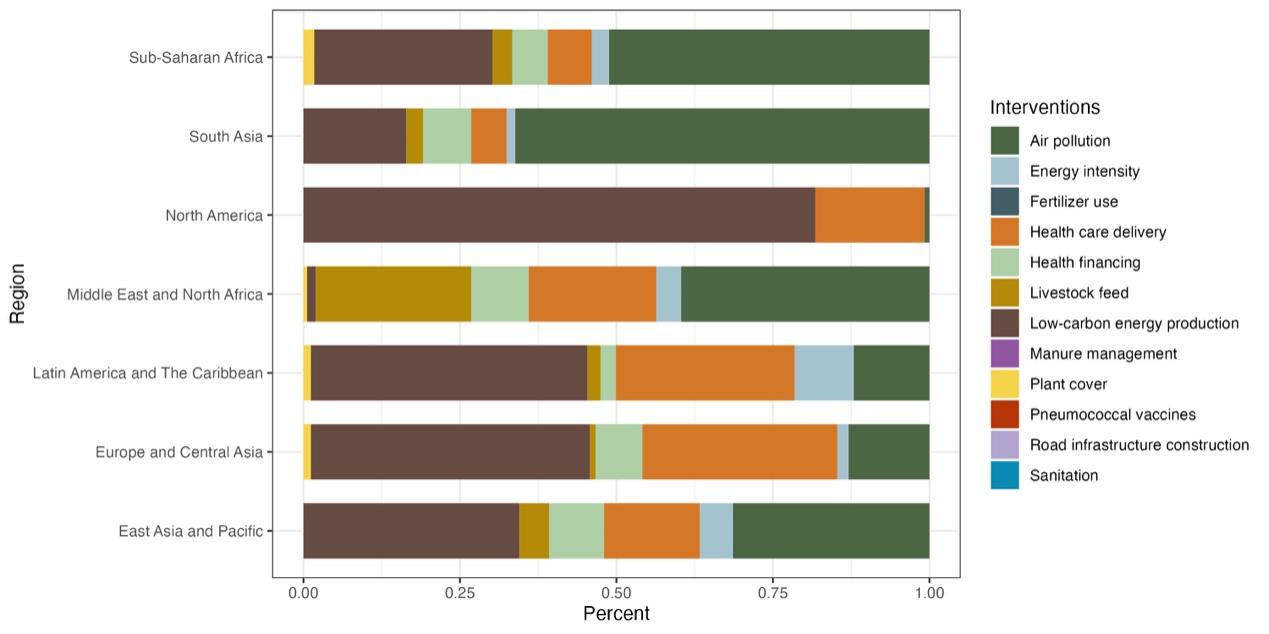


Fig.F.4. Breakdown of contributions of optimized decision-making packages to relieving HTD burden of metabolic diseases by regions.

Table F.4. Breakdown of contributions of optimized decision-making packages to relieving HTD burden of metabolic diseases by regions.

|  | East Asia and Pacific | Europe and Central Asia | Latin America and The Caribbean | Middle East and North Africa | North America | South Asia | Sub-Saharan Africa |
| --- | --- | --- | --- | --- | --- | --- | --- |
| Air pollution | 31.37% | 12.93% | 12.10% | 39.67% | 0.79% | 66.17% | 51.17% |
| Energy intensity | 5.32% | 1.79% | 9.43% | 3.96% | / | 1.37% | 2.76% |
| Fertilizer use | / | / | / | / | / | / | / |
| Health care Delivery | 15.29% | 31.15% | 28.55% | 20.40% | 17.43% | 5.66% | 7.06% |
| Health Financing | 8.77% | 7.48% | 2.45% | 9.18% | / | 7.67% | 5.66% |
| Livestock feed | 4.75% | 0.93% | 2.10% | 24.83% | / | 2.73% | 3.17% |
| Low-carbon energy production | 34.49% | 44.54% | 44.19% | 1.41% | 81.78% | 16.41% | 28.46% |
| Manure Management | / | / | / | / | / | / | / |
| Plant cover | / | 1.18% | 1.17% | 0.56% | / | / | 1.72% |
| Pneumococcal vaccines | / | / | / | / | / | / | / |
| Road Infrastructure construction | / | / | / | / | / | / | / |
| Sanitation | / | / | / | / | / | / | / |

1. Non-infectious Respiratory Diseases


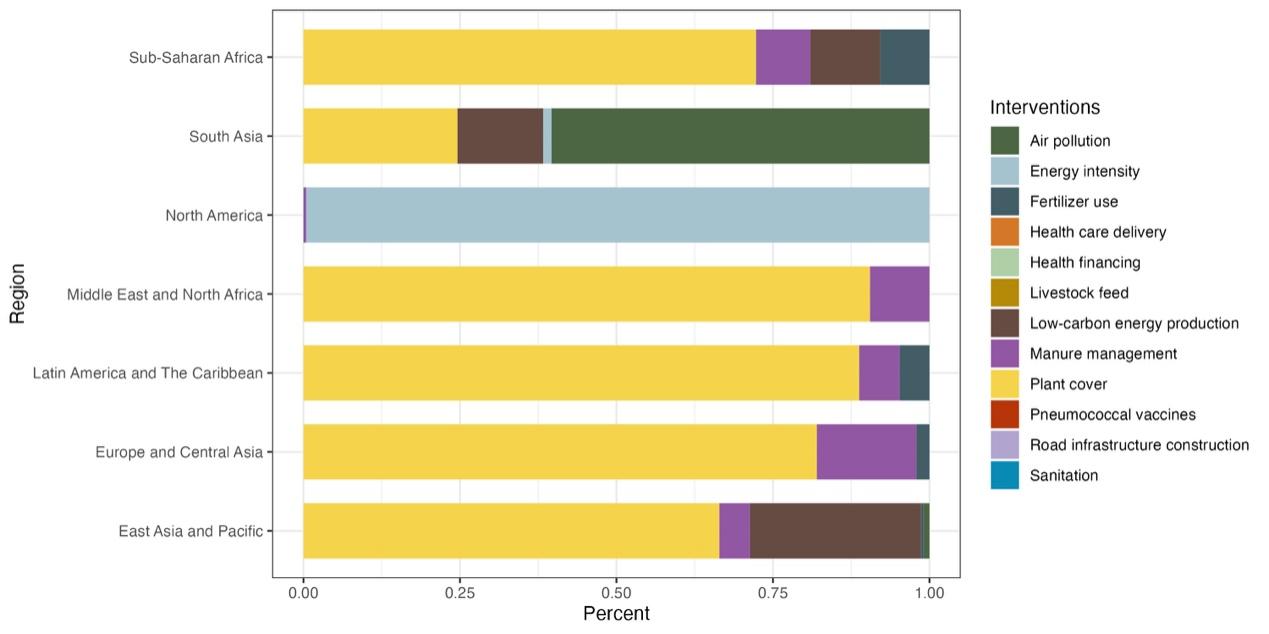


Fig.F.5. Breakdown of contributions of optimized decision-making packages to relieving HTD burden of non-infectious respiratory diseases by regions.

Table F.5. Breakdown of contributions of optimized decision-making packages to relieving HTD burden of non-infectious respiratory diseases by regions.

|  | East Asia and Pacific | Europe and Central Asia | Latin America and The Caribbean | Middle East and North Africa | North America | South Asia | Sub-Saharan Africa |
| --- | --- | --- | --- | --- | --- | --- | --- |
| Air pollution | 0.93% | / | / | / | / | 60.39% | / |
| Energy intensity | / | / | / | / | 99.57% | 1.32% | / |
| Fertilizer use | 0.41% | 2.10% | 4.78% | / | / | / | 7.88% |
| Health care Delivery | / | / | / | / | / | / | / |
| Health Financing | / | / | / | / | / | / | / |
| Livestock feed | / | / | / | / | / | / | / |
| Low-carbon energy production | 27.35% | / | / | / | / | 13.70% | 11.16% |
| Manure Management | 4.87% | 15.89% | 6.45% | 9.51% | 0.43% | / | 8.67% |
| Plant cover | 66.44% | 82.01% | 88.77% | 90.49% | / | 24.59% | 72.30% |
| Pneumococcal vaccines | / | / | / | / | / | / | / |
| Road Infrastructure construction | / | / | / | / | / | / | / |
| Sanitation | / | / | / | / | / | / | / |
