# Supplementary material for: Optimal decision-making in relieving global high temperature-related disease burden by data-driven simulation
Source: Infect Dis Model. 2024 Mar 19;9(2):618–33. doi: 10.1016/j.idm.2024.03.001 (PMC11026972; doi:10.1016/j.idm.2024.03.001)
Supplement: Multimedia component 7 [file mmc7.docx]

**Appendix G:Sensitivity Analysis**

The reduction value of disease burden gradually increases with the increase of intervention intensity, which can provide decision makers in different regions with appropriate intervention target levels.


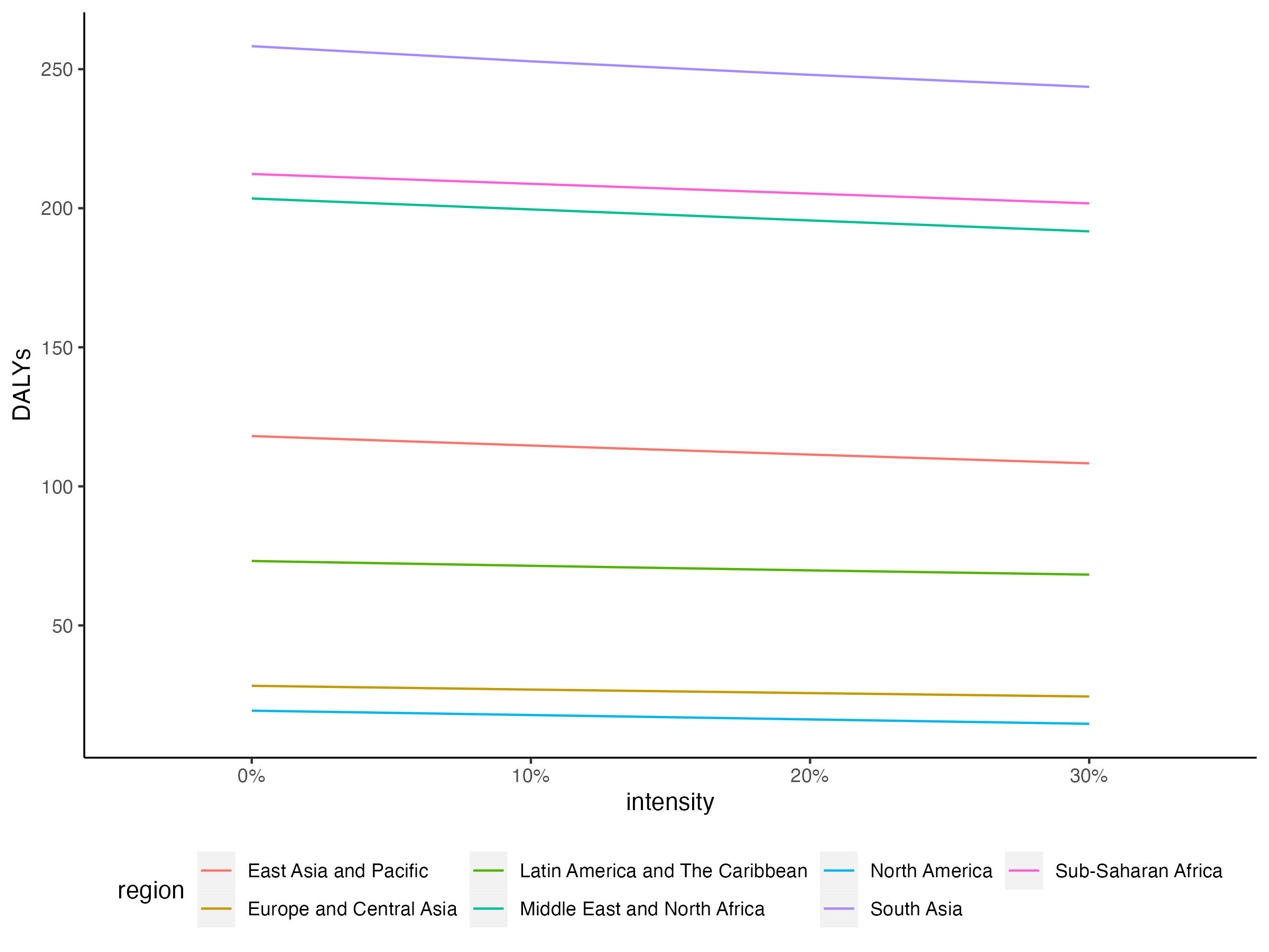


Fig.G.1. Impact of different intervention intensities on the reduction of high temperature-related disease burden in different regions in 2019
